# The Oncogenic Roles of *PTTG1* and *PTTG2* Genes and Pseudogene *PTTG3P* in Head and Neck Squamous Cell Carcinomas

**DOI:** 10.3390/diagnostics10080606

**Published:** 2020-08-18

**Authors:** Inga Grzechowiak, Justyna Graś, Dominika Szymańska, Martyna Biernacka, Kacper Guglas, Paulina Poter, Andrzej Mackiewicz, Tomasz Kolenda

**Affiliations:** 1 Department of Cancer Immunology, Chair of Medical Biotechnology, Poznan University of Medical Sciences, 8 Rokietnicka Street, 60-806 Poznan, Poland; grzechowiak.inga@gmail.com (I.G.); justyna.grass@gmail.com (J.G.); dominika040410@gmail.com (D.S.); martyna.biernacka20@gmail.com (M.B.); andrzej.mackiewicz@wco.pl (A.M.); 2 Laboratory of Cancer Genetics, 15 Garbary Street, 61-866 Poznan, Poland; kacper.guglas@gmail.com; 3 Postgraduate School of Molecular Medicine, Medical University of Warsaw, 61 Zwirki i Wigury Street, 02-091 Warsaw, Poland; 4 Department of Oncologic Pathology and Prophylaxis, Poznan University of Medical Sciences, Greater Poland Cancer Center, 15 Garbary Street, 61-866 Poznan, Poland; paulina.poter@gmail.com; 5 Department of Pathology, Pomeranian Medical University, 1 Unii Lubelskiej Street, 71-242 Szczecin, Poland; 6 Department of Diagnostics and Cancer Immunology, Greater Poland Cancer Centre, 15 Garbary Street, 61-866 Poznan, Poland

**Keywords:** PTTG3P, PTTG1, PTTG2, HNSCC, TCGA, biomarker, pseudogene, non-coding RNA, TP53

## Abstract

Background: Head and neck squamous cell carcinomas are a group of heterogeneous diseases that occur in the mouth, pharynx and larynx and are characterized by poor prognosis. A low overall survival rate leads to a need to develop biomarkers for early head and neck squamous cell carcinomas detection, accurate prognosis and appropriate selection of therapy. Therefore, in this paper, we investigate the biological role of the *PTTG3P* pseudogene and associated genes *PTTG1* and *PTTG2* and their potential use as biomarkers. Methods: Based on TCGA data and the UALCAN database, *PTTG3P*, *PTTG1* and *PTTG2* expression profiles and clinicopathological features with TP53 gene status as well as expression levels of correlated genes were analyzed in patients’ tissue samples. The selected genes were classified according to their biological function using the PANTHER tool. Gene Set Enrichment Analysis software was used for functional enrichment analysis. All statistical analyses were performed using GraphPad Prism 5. Results: In head and neck squamous cell carcinomas, significant up-regulation of the *PTTG3P* pseudogene, *PTTG1* and *PTTG2* genes’ expression between normal and cancer samples were observed. Moreover, the expression of *PTTG3P*, *PTTG1* and *PTTG2* depends on the type of mutation in TP53 gene, and they correlate with genes from p53 pathway. *PTTG3P* expression was significantly correlated with *PTTG1* as well as *PTTG2*, as was *PTTG1* expression with *PTTG2*. Significant differences between expression levels of *PTTG3P*, *PTTG1* and *PTTG2* in head and neck squamous cell carcinomas patients were also observed in clinicopathological contexts. The contexts taken into consideration included: T-stage for *PTTG3P*; grade for *PTTG3*, *PTTG1* and *PTTG2*; perineural invasion and lymph node neck dissection for *PTTG1* and HPV p16 status for *PTTG3P*, *PTTG1* and *PTTG2*. A significantly longer disease-free survival for patients with low expressions of *PTTG3P* and *PTTG2*, as compared to high expression groups, was also observed. Gene Set Enrichment Analysis indicated that the *PTTG3* high-expressing group of patients have the most deregulated genes connected with DNA repair, oxidative phosphorylation and peroxisome pathways. For *PTTG1*, altered genes are from DNA repair groups, Myc targets, E2F targets and oxidative phosphorylation pathways, while for *PTTG2*, changes in E2F targets, G2M checkpoints and oxidative phosphorylation pathways are indicated. Conclusions: *PTTG3P* and *PTTG2* can be used as a prognostic biomarker in head and neck squamous cell carcinomas diagnostics. Moreover, patients with high expressions of *PTTG3P*, *PTTG1* or *PTTG2* have worse outcomes due to upregulation of oncogenic pathways and more aggressive phenotypes.

## 1. Introduction

Head and neck squamous cell carcinomas (HNSCCs) belong to the most prevalent cancers, with an annual incidence of 600,000 cases [1]. These cancers are anatomically located in the oral cavity, the nasal cavity, the nasopharynx, the oropharynx, paranasal sinuses, the hypopharynx and the larynx. HNSCCs develop mostly via chemical carcinogenesis, and the main risk factors are alcohol, tobacco use and human papillomavirus (HPV) infections [1,2,3]. Treatments include surgical eradication, radiotherapy (RT) and chemotherapy (CT), but they are associated with a reduction in life expectancy and quality of life. There is an urgent need for more effective therapies to improve treatment outcomes [4].

The main cause of death is metastasis, which in HNSCC is most commonly formed in the cervical lymph nodes. The absence of clinical symptoms makes it impossible to detect this type of cancer in the early stages of the disease. Molecular markers such as cell cycle regulators, cell adhesion proteins and growth factors for cancer metastasis have recently been studied [5].

The latest class of studied biomarkers are non-coding RNAs (ncRNAs), which can be distinguished into two main groups: short RNAs (e.g., miRNAs) and long RNAs (lncRNAs). These molecules are well known elements of the competitive endogenous RNA (ceRNA) network. However, recent studies revealed new elements of the ceRNA network, which are RNA pseudogene transcripts [6].

Pseudogenes are relicts of parental genes, which have lost their original functions through accumulation of mutations. The aforementioned functions include the capability to synthesize proteins due to events such as insertions, premature stop codons, frameshift-causing deletions and splicing errors [7]. However, reports indicate that they have defined roles in the regulation of gene expression [6,8]. Based on the structure and origin of pseudogenes, they were divided into two groups, processed and unprocessed transcripts, which contain introns [8]. Pseudogenes and corresponding protein-coding genes can talk with each other, pseudogene 1 (*PTENp1*) being a notable example, as it contains elements of the miRNA response (MRE) shared by the corresponding gene encoding protein, *PTEN*. Thus, the pseudogene and the coding gene compete for the same miRNAs [7]. It was indicated that *PTENp1* is involved in oral squamous cell carcinoma (OSCC) [9]. *PTENp1* acts as a potential tumor suppressor due to the reduction of its copy number, independent of *PTEN*. It has also shown examples of ectopic expression of *PTENP1* inhibited proliferation, colony formation and migration of HNSCC cells, which proves that *PTENP1* may serve as a prognostic factor in patients with HNSCC [10].

Recently, the pituitary tumor processing gene (*PTTG*) family was described in the context of carcinogenesis. The *PTTG* family includes two genes with protein products *PTTG1*, *PTTG2* and one processed pseudogene, *PTTG3P* [11]. The protein product of the pituitary tumor processing gene (*PTTG*) was isolated for the first time from a rat’s pituitary gland tumor cells and shown to be transforming in vitro and tumorigenic in vivo [12]. It was demonstrated that the pituitary tumor transforming gene (*PTTG*) plays a role in tumor initiation and progression, including mitosis control, cell transformation and DNA repair [13]. The first one, *PTTG1* regulator of sister chromatid separation, securin (previously named as pituitary tumor-transforming gene 1-*PTTG1* or tumor-transforming protein 1-*TUTR1*) is a member of the family of securin proteins. *PTTG1* displays many biological functions such as DNA repair, the inhibition of sister chromatid separation, organ development and the regulation of expression and secretion of angiogenic and metastatic factors [14]. The second member of the *PTTG* family is the pituitary tumor-transforming gene 2 (*PTTG2*). *PTTG2* is considered to be a regulator of the cell circadian clock, functioning in cell mitosis, differentiation and apoptosis [15]. The latter is a pituitary tumor-transforming 3 pseudogene (*PTTG3P*; previous symbol: *PTTG3*). It is an intronless gene highly homologous to *PTTG1* and *PTTG2* [16]. *PTTG3P* can be expressed in a variety of normal adult tissues [16]. Moreover, it has a limited protein-coding capacity, and thus, it is regarded as one long non-coding RNA (lncRNA). The mechanisms of *PTTG3P* functions are not yet well understood, but it has been demonstrated that *PTTG3P* promotes both proliferation and invasion throght upregulation of *PTTG1* and is an indicator of bad prognosis in cervical cancer and hepatocellular carcinoma [17,18].

It should be noted that the PTTG family interacts with p53. For example, PTTG1 is thought to influence p53 activity by blocking p53 binding to DNA [19]. Moreover, Méndez-Vidal et al. indicated that *PTTG2* silencing results in increased levels of p53 protein [20]. Suppressor p53 serves an important role in the cellular homeostasis maintenance [21]. Mutations in TP53 gene are prevalent in many cancers, including HNSCC [21,22]. Studies suggest that evaluation of TP53 mutation type may have a prognostic value and could prove useful in predicting response to radio- and chemotherapy as well as become a potential target for new therapeutic strategies [22]. Mutations in p53 can be divided into two main groups: DNA contact mutations (such as R273H and R280K) and conformational mutations (such as R175H and V143A). Missense mutations in the DNA-binding domain are the most common ones among TP53 mutations [22]. It should be noted that besides loss-of-function of wild type p53 and dominant-negative forms of p53, some mutations cause gain-of-function and lead to tumor progression, metastatic potential as well as may influence drug resistance [23]. Recently, Caponio et al. confirmed that classification of HNSCC patients based on TP53 mutation types serves as an independent prognostic factor. Moreover, specific TP53 mutations are associated with different anatomical sites of HNSCC and modulate its cellular activity in different ways [24].

In this study we examined the expression levels of *PTTG3P*, *PTTG1* and *PTTG2* in HNSCC using data from The Cancer Genome Atlas (TCGA), making it the first comprehensive study of its kind based on a large group of patients. The role of these genes in HNSCC biology and their potential as biomarkers in clinical practice was investigated.

## 2. Materials and Methods

### 2.1. TCGA Data

The TCGA expression data of *PTTG3P*, *PTTG1* and *PTTG2*, as well as the expression of selected genes and clinical data were downloaded from cBioPortal (Head and Neck Squamous Cell Carcinoma, TCGA) [25] and from UALCAN databases (http://ualcan.path.uab.edu) [26]. All data is available online; access is unrestricted and does not require patient’s consent or other permissions. The use of the data does not violate the rights of any person or any institution.

### 2.2. Data Analysis

The expression levels of *PTTG3P*, *PTTG1* and *PTTG2* genes were analyzed and correlated with clinicopathological parameters such as: age (≤60 vs. >60), gender (women vs. men), smoking category (1, 2 vs. 3, 4, 5), alcohol history (negative vs. positive), T-stage (T1 + T2 vs. T3 + T4), N-stage (N0 + N1 vs. N2 + N3), cancer grade (G1 + G2 vs. G3 + G4), cancer stage (I + II vs. III + IV), HPV p16 marker (negative vs. positive), perineural invasion (negative vs. positive), lymphovascular invasion (negative vs. positive), and lymphoid neck dissection status (negative vs. positive) in all localizations of the HNSCC samples. Next, in a group of 522 patients with adequate gene expression, disease-free survival (DFS) and overall survival (OS) data were selected for analysis. DFS and OS probability were estimated in patients with high and low expression of *PTTG3P*, *PTTG1* or *PTTG2*.

### 2.3. Gene Analysis

Association between *PTTG3P*, *PTTG1* and *PTTG2* expression levels and TP53 was analyzed using UALCAN database. Next, specific mutations in TP53, described previously by Caponio et al. as well as Freed-Pastor and Prives [23,24], and expression levels of *PTTG3P*, *PTTG1* and *PTTG2* were analyzed using TCGA data. The mutant p53-interacting partners and genes transcriptionally activated by mutant p53 protein, described by Freed-Pastor and Prives [23], and their correlations with *PTTG3P*, *PTTG1* or *PTTG2* were evaluated using StarBase v3.0 database [27].

Genes positively and negatively correlated with *PTTG3P*, *PTTG1* and *PTTG2* (Spearman’s correlation *R* > 0.3 or *R* < −0.3, respectively) were obtained from cBioportal and analyzed using the REACTOME pathway database [28].

### 2.4. Functional Enrichment Analysis and Prediction of Gene Function

Gene Set Enrichment Analysis (GSEA) software version 3.0 (http://www.gsea-msigdb.org/gsea/index.jsp) was used for the aforementioned analysis of functional enrichment [29]. HNSCC patients were divided into two groups with high and low expressions of *PTTG3P*, *PTTG1* and *PTTG2* (groups divided by the mean of the expression level). The input file contained expression data for 20,530 genes and 565 patients. We used 1000 gene set permutations for the analysis and pathway (hallmark gene sets (H) and collection from MSigDB) with nominal *p*-value *p* ≤ 0.05 and FDR ≤ 0.25 were considered significant. Next, the interactions between protein encoding genes in the pathway, which were most significantly enriched in a group of patients with high expression of *PTTG3P*, *PTTG1* and *PTTG2* were analyzed using the GeneMANIA prediction tool (http://genemania.org) [30].

### 2.5. Statistical Analysis

All statistical analyses were performed using GraphPad Prism 5 (GraphPad, San Diego, CA, USA). The Shapiro–Wilk normality test, *t*-test and Mann–Whitney *U* test were used for measuring *PTTG3P*, *PTTG1* and *PTTG2* levels (depending on clinical parameters) and gene expressions (depending on *PTTG3P*, *PTTG1* and *PTTG2* subgroups). The expression levels of *PTTG3P*, *PTTG1* and *PTTG2* (depending on the cancer location) were compared using one-way ANOVA obtained using Dunn’s multiple test. All TCGA data are presented as a median with SEM. For disease-free survival (DSF) and overall survival (OS) prognosis, the Log-Rank (Mantel–Cox) and Gehan–Breslow–Wilcoxon tests were used. The Hazard Ratio (Mantel–Haenszel; HR) and 95% Confidence Interval (CI) of ratio were calculated. In all analyses, *p* < 0.05 was used to determine statistical significance.

### 2.6. Availability of Data and Materials

The datasets used and/or analyzed during the current study are available from the corresponding author on reasonable request. Raw data is available on the cBioPortal, UALCAN and StarBase v3.0 databases.

## 3. Results

### 3.1. PTTG3P, PTTG1 and PTTG2 Are Upregulated in Most Cancers, Including HNSCC

**Figure 1:**
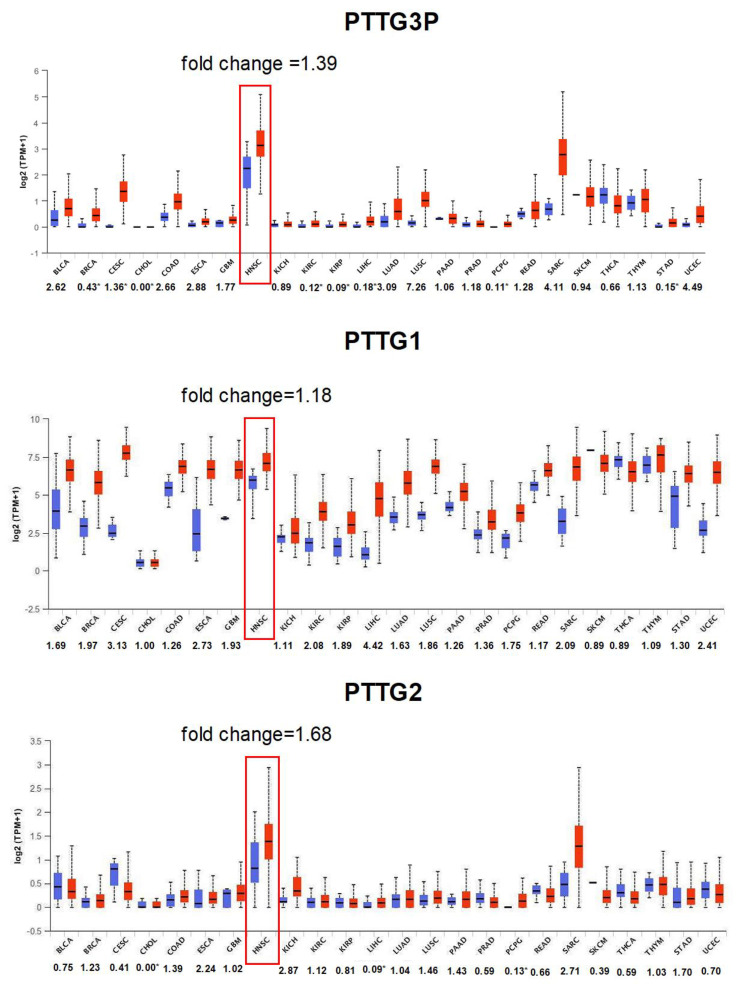
The expression levels of *PTTG3P*, *PTTG1* and *PTTG2* in HNSCC patients. Expression of *PTTG3P*, *PTTG1* and *PTTG2* in normal and cancer tissues. Graphs from the UALCAN database; modified; fold change for *PTTG3P*, *PTTG1* and *PTTG2* was calculated between normal (blue boxes) and cancer tissue (red boxes) and placed next to the cancer acronyms (see abbreviation section). The changed fold values marked with an asterisk are values for which the median value of healthy tissues was equal to zero. In this case, the median value in cancer tissues was given as the fold change value.

Firstly, the expression levels of *PTTG3P* and two other members of the *PTTG* family, *PTTG1* and *PTTG2*, were analyzed across twenty-four different cancers, including squamous cell carcinomas, adenocarcinomas and other cancers based on data from the UALCAN database. In the group of squamous cell carcinomas, the highest fold change of *PTTG3P* was observed for LUSC (7.26), while the lowest for CESC (1.36); For HNSCC, a 1.39-fold change was indicated. In the same group of cancers, the highest fold change of *PTTG1* was observed for CESC (3.14) and the lowest for HNSC (1.18). For *PTTG2*, the highest fold change value was observed in HNSC (1.68), while the lowest in CESC (0.41) in squamous cell carcinomas. In the group of adenocarcinomas, the highest fold change of *PTTG3P* was indicated for LUAD (3.09) and the lowest for STAD (0.15). In the same group of cancers, the highest fold change of PTTG1 was observed for LUAD (1.63), while the lowest for READ (1.17). For *PTTG2*, the highest fold change value in this group of cancer was obtained for STAD (1.70) and the lowest for PRAD (0.59). In the group of other cancers, the highest fold change of *PTTG3P* was observed for UCEC (4.49) and the lowest for KIRP (0.09). In the same group of cancers, the highest fold change of *PTTG1* was obtained for LIHC (4.42) and the lowest for SKCM (0.89) and THCA (0.89). For *PTTG2* the highest fold change value in this group of cancer was obtained for KICH (2.87) and the lowest for LIHL (0.09). All obtained results are presented in Figure 1.

**Figure 2:**
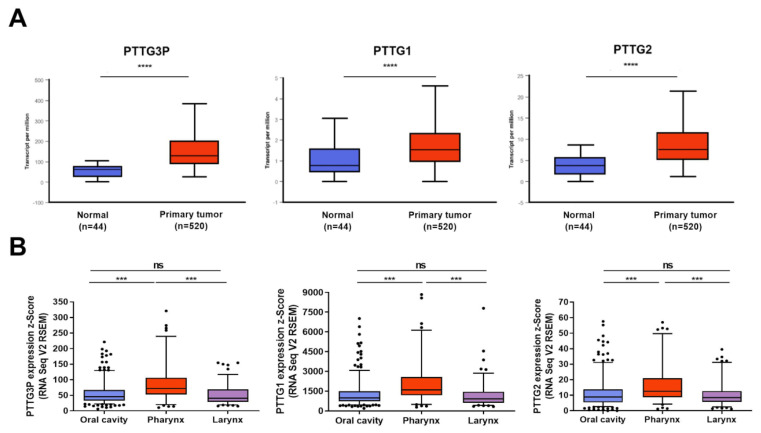
The expression levels of *PTTG3P*, *PTTG1* and *PTTG2* in HNSCC patients. Expression of *PTTG3P*, *PTTG1* and *PTTG2* in normal and cancer tissues (A); expression of *PTTG3P*, *PTTG1* and *PTTG2* depending on HNSCC localization (**B**). Graphs from UALCAN database; modified (**A**); unpaired *t*-test; the graphs show the median of value presented as transcripts per million; and box and whiskers with 5–95 percentile, one-way ANOVA obtained using Dunn’s multiple comparisons tests (**B**). ns—not significant (*p* > 0.05), **** *p* ≤ 0.0001; *** *p* ≤ 0.001; *p* < 0.05 considered as significant.

In HNSCC, significant up-regulation of *PTTG3P*, *PTTG1* and *PTTG2* expressions between normal and cancer samples was observed (3.758 vs. 7.534; *p* = 1.62 × 10^−12^), (62.059 vs. 129.031; *p* = 1.62 × 10^−12^) and (0.776 vs. 1.533; *p* = 2.04 × 10^−12^), respectively, Figure 2A. Moreover, based on the National Institute of Health’s (NIH) classification, HNSCC patients were divided into three groups according to the localization of cancer and expression levels of *PTTG3P*, *PTTG1* and *PTTG2*. Afterwards, these subgroups were analyzed. Differences were not observed between tumors from the oral cavity and larynx in all the analyzed genes (*p* > 0.05). Statistical significance was obtained between tumors from the oral cavity and pharynx and between tumors from the pharynx and larynx in all three genes (*p* < 0.001), Figure 2B.

### 3.2. PTTG3P Positively Correlates with PTTG1 and PTTG2 in HNSCC Patients

**Figure 3:**
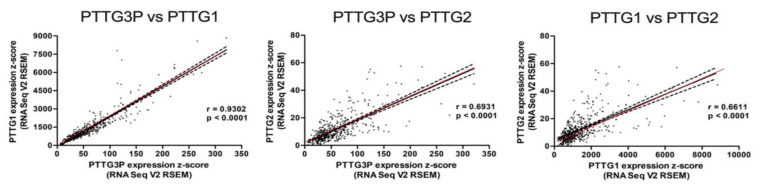
Correlation between expression levels of *PTTG3P* and *PTTG1*, *PTTG3P* and *PTTG2*, and *PTTG1* and *PTTG2* in HNSCC patients; Spearman correlation test with *p* < 0.05 considered as significant.

To analyze the correlation between *PTTG3P* and *PTTG1* or *PTTG2* gene expressions in HNSCC samples, the Spearman test was used. The results showed that *PTTG3P* expression was positively correlated with *PTTG1* (*r* = 0.9302, *p* < 0.0001) as well as *PTTG2* (*r* = 0.6931, *p* < 0.0001) and also the *PTTG1* expression was positively correlated with *PTTG2* (*r* = 0.6611, *p* < 0.0001). All results are shown in Figure 3 below.

### 3.3. Expression Levels of PTTG3P, PTTG1 and PTTG2 Depend on TP53 Status and Correlate with Expression of the Genes from the p53 Pathway

**Figure 4:**
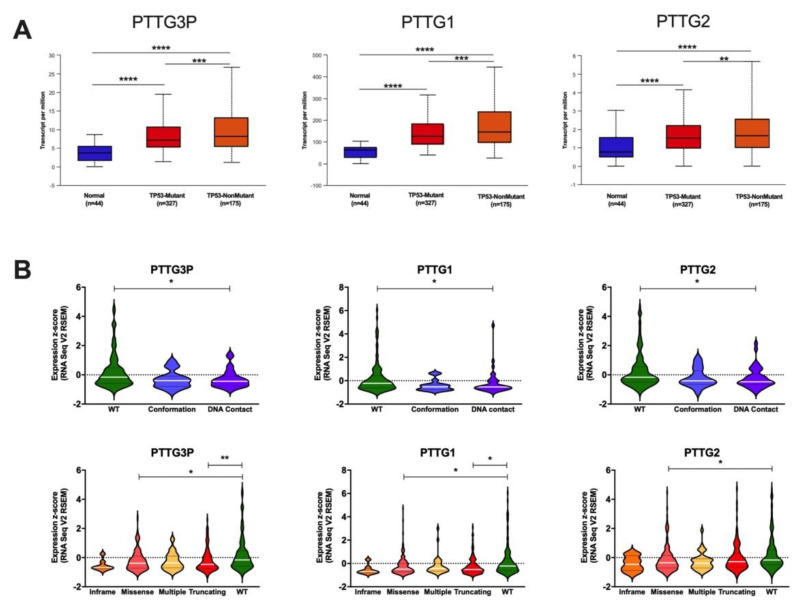
Expression level of *PTTG3P*, *PTTG1* and *PTTG2* depending on the TP53 status (**A**) and depending on the specific type of TP53 mutations (**B**) in HNSCC patients; graphs from UALCAN database included in panel A; modified, **** *p* ≤ 0.0001; *** *p* ≤ 0.001; ** *p* < 0.01; * *p* < 0.05.

Due to TP53 status being an important marker for HNSCC, the dependent expression levels of *PTTG3P*, *PTTG1* and *PTTG2* were checked. In all analyzed genes, a significantly higher (*p* < 0.05) expression of mutant and wild type TP53 cases (compared to normal samples) was observed. Moreover, expression levels of *PTTG3P*, *PTTG1* and *PTTG2* were significantly higher in patients with non-mutated as compared to mutated TP53 genes (*p* = 2.20 × 10^−5^; *p* = 1.67 × 10^−5^ and *p* = 1.73 × 10^−3^, respectively), Figure 4A. To further analyze the connection between *PTTG3P*, *PTTG1* and *PTTG2* expression and TP53 status, the expression levels of PTTG gene family were checked in groups of patients divided depending on TP53 gene mutation type. In all examined cases, the highest expressions of *PTTG3P*, *PTTG1* and *PTTG2* were observed in wild type TP53 versus DNA contact mutations (*p* = 0.0127; *p* = 0.0072; *p =* 0.0323, respectively), Figure 4B. Analysis of different division of TP53 mutation types showed significant differences between missense mutations versus wild type and truncating mutations versus wild type for *PTTG3P* and *PTTG1* (*p* = 0.0011; *p* = 0.0025, respectively). Finally, for *PTTG2*, significant difference was observed only between missense mutations versus wild type TP53 (*p* = 0.0099), Figure 4B.

**Figure 5:**
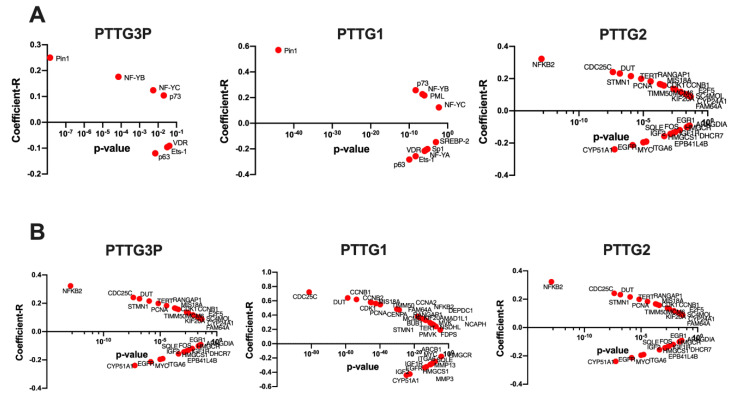
Correlation of *PTTG3P, PTTG1* or *PTTG2* with mutant p53-interacting partners (**A**) and transcriptionally activated genes by mutant p53 protein (**B**). Genes only significant (*p* < 0.05) and in panel B with correlation *R* < −0.17 and *R* > 0.17 were indicated.

Next, selected mutant p53-interacting partners and genes transcriptionally activated by mutant p53 protein were analyzed. In the case of mutant p53-interacting partners and *PTTG3P* statistically significant positive correlation (*p* < 0.05) of NF-YB, NF-YC, Pin1 and p73 expression, and negative of Ets-1, VDR and p63 was indicated. Positive correlation was also found between *PTTG1* and NF-YB, NF-YC, Pin1, PML and p73, in contrast to NF-YA, Sp1, Ets-1, VDR, SREBP-2 and p63, which were negatively correlated. The expression of last examined gene, *PTTG2*, was positively correlated with NF-YB, Pin1, PML and p73 and significantly negatively correlated only with NF-YA and p63 (Figure 5A). However, observed coefficient-R was not strong for analyzed genes (Supplementary Table S1).

The group of genes transcriptionally activated by mutant p53 protein and positively correlated (*R* > 0.2, *p* < 0.05) with *PTTG3P* comprised CCNA2, KIF20A, MCM6, TIMM50, TERT, CENPA, CCNB2, CDK1, CCNB1, DUT, MIS18A, STMN1 and CDC25C, whereas in the group of negatively correlated (*R* < −0.17, *p* < 0.05) were IGF2, MMP3, CYP51A1 and MMP13. In the group of genes positively correlated *(R* > 0.2, *p* < 0.05) with *PTTG1* were NCAPH, NSDHL, DEPDC1, BUB1, MVK, MAD1L1, TERT, PMVK, KIF20A, MCM6, CCNA2, NFKB2, RANGAP1, FAM64A, CENPA, TIMM50, MIS18A, PCNA, CDK1, STMN1, CCNB2, CCNB1, DUT and CDC25C, in contrast to the negatively correlated genes (*R* < −0.17, *p* < 0.05): MYC, ABCB1, HMGCR, SQLE, MMP13, ITGA6, MMP3, HMGCS1, IGF1R, EGFR, IGF2 and CYP51A1. *PTTG2* was found to be positively correlated (*R* > 0.2, *p* < 0.05) with STMN1, DUT, CDC25C and NFKB2 and negatively correlated (*R* < −0.17, *p* < 0.05) with ITGA6, MYC, EGFR and CYP51A1. All data is presented in Figure 5B and Supplementary Table S2.

It should be noted that statistically significant (*p* < 0.05) relationship between the expression levels and patients’ survival was observed only in the case of: Pin1, p73, IGF1R, ABCB1 and SQLE, (data from StarBase v3.0); Supplementary Tables S3 and S4.

### 3.4. PTTG3P, PTTG1 and PTTG2 Levels Differ Depending on Clinicopathological Parameters

The expression levels of *PTTG3P*, *PTTG1* and *PTTG2* were analyzed in the context of clinicopathological parameters in HNSCC patients. Significant differences between expression levels of *PTTG3P*, *PTTG1* and *PTTG2* were observed with regard to T-stage (*p* = 0.037 for *PTTG3P*), grade (*p* < 0.0001 for *PTTG3* and *PTTG1*, *p* = 0.005 for *PTTG2*), perineural invasion (*p* = 0.032 for *PTTG1*), lymph node neck dissection (*p* = 0.033 for *PTTG1*) and HPV p16 status (*p* < 0.0001 for *PTTG3P*, *PTTG1* and *PTTG2*). All data are summarized in Table 1.

### 3.5. Patients with Low PTTG3P and PTTG2 Expression Display Longer Disease-Free Survival

**Figure 6:**
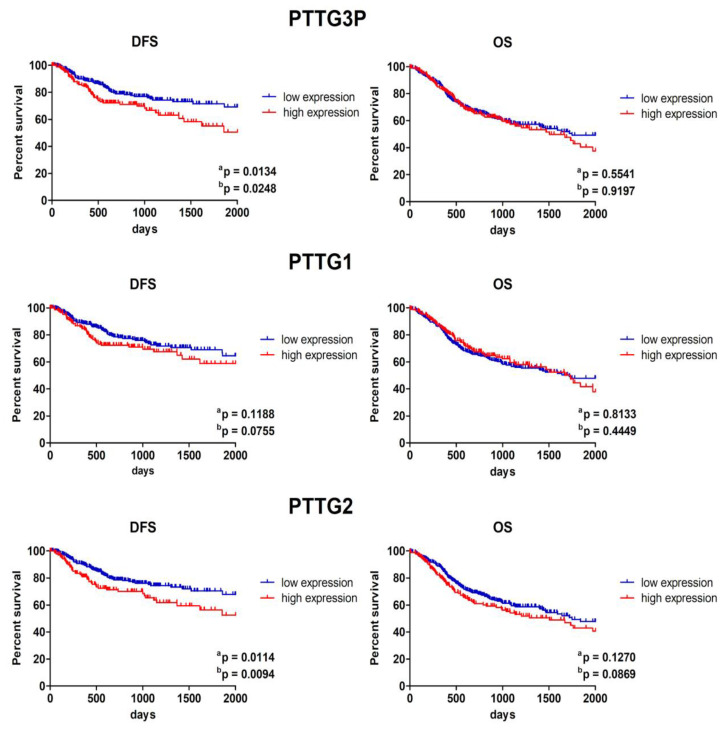
Disease-free survival (DFS) and overall survival (OS) of HNSCC patients with all localizations of tumors depending on the *PTTG3P*, *PTTG1* or *PTTG2* expression levels; results presented for 5.5 years of observation; low and high subgroups of patients divided based on median expression; a—Log-rank (Mantel–Cox) test, b—Gehan–Breslow–Wilcoxon test; *p* < 0.05 considered as significant.

HNSCC samples were divided into low and high *PTTG3P*, *PTTG1* or *PTTG2* expression groups based on the median expression level of the selected gene and DFS, as well as OS between groups of patients were compared. Significantly longer DFS of patients with low expressions of *PTTG3P* and *PTTG2* compared to high expression groups (*p* = 0.0094 and *p* = 0.0248, respectively) was found. No statistical significance was discovered in the length of DFS between *PTTG1* low and high expression groups. No notable differences in the length of OS between low and high expression groups in any of the studied genes were detected, Figure 6.

### 3.6. Expression Levels of PTTG3P, PTTG1 and PTTG2 Correlate with Genes Involved in Important Cellular Processes

Next, positive (*R* > 0.3) and negative (*R* < −0.3) Pearson correlations of *PTTG3P*, *PTTG1* and *PTTG2* with genes involved in important cellular processes were analyzed using REACTOME pathway analysis. For *PTTG3P*, 1252 positively correlated genes are mostly involved in cell cycle, gene expression, metabolism of RNA, DNA repair, DNA replication, organelle biogenesis and maintenance, chromatin organization, programmed cell death as well as in HIV viral infection. In spite of this, only 46 negatively correlated genes were indicated and connected with developmental biology processes (keratinization) and signal transduction.

**Figure 7:**
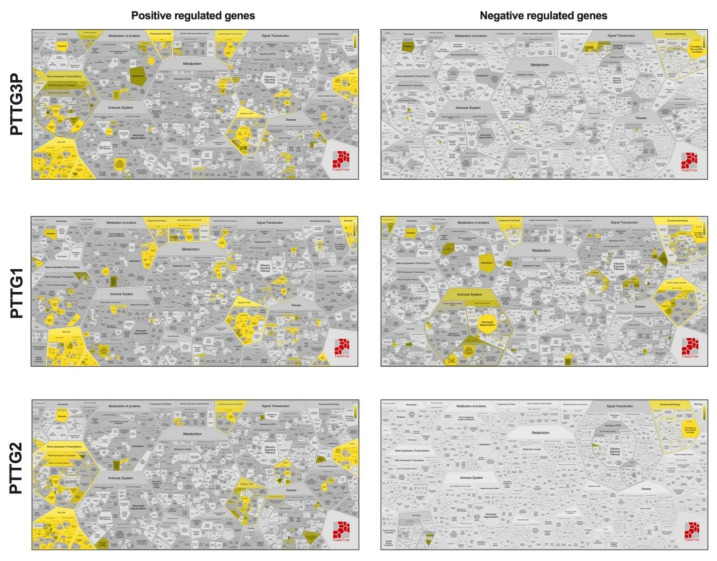
Positive and negative correlation of *PTTG3P*, *PTTG1* and *PTTG2* with genes involved in important cellular processes. Only genes with Spearman’s correlation *R* > 0.3, *R* < −0.3 and *p* < 0.05 were indicated in REACTOME pathway analysis as yellow fields in FoamTree graphs.

For *PTTG1*, 1277 positively correlated genes connected with cell cycle, the metabolism of RNA, cellular responses to external stimuli, DNA repair, DNA replication, programmed cell death and protein localization as well as viral infection (HIV and influenza) were indicated. The analysis for 264 negatively regulated genes for *PTTG1* indicated that they are involved in the immune system, developmental biology, vesicle-mediated transport, programmed cell death and cell–cell communication. The last gene analyzed was *PTTG2*. It had 593 positively correlated genes found in processes such as cell cycle, gene expression (transcription), DNA repair, metabolism of RNA, DNA replication as well as in organelle biogenesis and maintenance. Surprisingly, only 2 genes were negatively correlated with *PTTG2* and associated with developmental biology processes (keratinization). All results are presented in Figure 7 and a list of genes in Supplementary Table S5.

### 3.7. Patients with High and Low Expressions of PTTG3P, PTTG1 or PTTG2 Have Different Patterns of Genes

**Figure 8:**
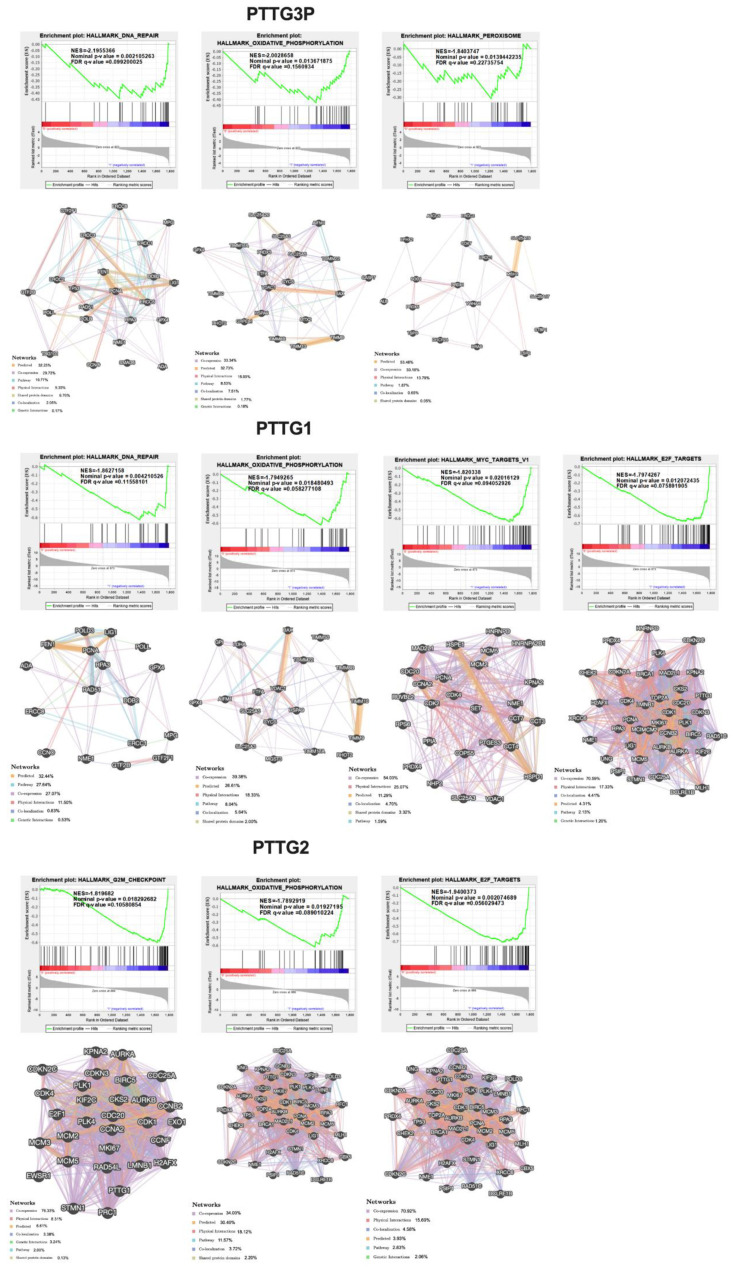
GSEA results for HNSCC patients analyzed in groups with low/high expression of *PTTG3P, PTTG1* and *PTTG2.* GSEA plots of significantly enriched datasets; NES (normalized enrichment score), *p*-value (nominal *p*-value), FDR q-value (false discovery rate). Normalized enrichment scores for GSEA analysis of MSigDB gene sets enriched in low (RED—0 on the graphs) and in high (VIOLET—1 on the graphs) *PTTG3P, PTTG1* and *PTTG2* patients. Interactions between protein coding genes in the pathway, which were the most significantly enriched in a group of patients with high expression of analyzed genes. Only results set with *p* ≤ 0.05 and FDR ≤ 0.25 were shown.

The functional implications of *PTTG3P*, *PTTG1* or *PTTG2* expression signatures were analyzed using gene set enrichment analysis (GSEA), and several top enriched datasets are shown in Figure 5. It was found that the most deregulated genes in *PTTG3P* high-expressing group of patients are clustered most significantly in DNA repair, oxidative phosphorylation and peroxisome pathways, NES = −2.196, NES = −2.003 and NES = −1.840, respectively. In the case of high-expressing group of patients in *PTTG1,* a gene set enrichment analysis indicated that the most deregulated genes in this group of patients are clustered most significantly in DNA repair, Myc targets, E2F targets and oxidative phosphorylation pathways, NES = −1.863, NES = −1.820, NES = −1.797 and NES = −1.794, respectively. In the *PTTG2* high-expressing group, gene set enrichment analysis indicated that the most deregulated genes in this group of patients are clustered most significantly in E2F targets, G2M checkpoints and oxidative phosphorylation pathways, NES = −1.940, NES = −1.820 and NES = −1.789, respectively; Figure 8 and list of genes are shown in Supplementary Table S6.

## 4. Discussion

The *PTTG* gene family consists of three members: one pseudogene (*PTTG3P*) and two protein coding genes, namely *PTTG1* and *PTTG2* [12]. Numerous studies have proven the significant role of *PTTGs* in cancer initiation and progression [11,13,16,17,18], although the mechanisms of these processes mostly remain unclear. It is known that *PTTG3P* influences tumor growth and metastasis in both cervical cancer and hepatocellular carcinoma [17,18]. Similarly, the second member, *PTTG1*, has been reported to promote migration and invasion in non-small cell lung cancer [11]. It has also been shown to play the role of an inflammation-related oncogene in hepatocellular carcinoma [31] Additionally, *PTTG1* was found to induce epithelial–mesenchymal transition in colon carcinoma [32].

To the best of our knowledge, this is the first study to present a comprehensive analysis of all three members of the *PTTG* gene family in HNSCC carcinoma patients. Based on TCGA data, we found that in HNSCC *PTTG3P* alteration is mild and is estimated to be around 1.39, compared to the highest fold change of *PTTG3P* observed in lung squamous cell carcinoma (LUSC), 7.26-fold change.

However, the expression of *PTTG3P* is significantly upregulated in HNSCC tumor tissues compared with normal samples. Moreover, the highest upregulation of *PTTG3P* expression was observed in pharynx tumors compared to oral and larynx locations, and instances of *PTTG1* and *PTTG2* were significantly upregulated in HNSCC tumor tissues compared with normal samples. As was the case with *PTTG3P*, the highest upregulation was observed in pharynx tumors compared to oral and larynx locations. This may indicate the significant role of these transcripts in pharyngeal tumors, which are known to be HPV dependent and, due to this fact, display distinct biological and clinical characteristics from HPV negative tumors [2].

As previously described, we observed differences in the expression of analyzed genes depending on TP53 status. TCGA data analysis of breast cancer patients performed by Lou et al. showed a positive correlation between the *PTTG3P* pseudogene and TP53 signaling pathways [33]. Moreover, Read et al. reported that *PTTG1* and *PBF* (*PTTG1IP*, PTTG1 interacting protein) together modulate the interaction with TP53, reduce p53 protein stability and affect its downstream targets which lead to worse outcomes in HNSCC patients [34]. Our results indicated that expression levels of *PTTG3P*, *PTTG1* and *PTTG2* depend on TP53 mutation type. Furthermore, they correlate with expression of the genes from the p53 pathway. We found that mutant p53-interacting partners such as: NF-YB, NF-YC, Pin1, PML and p73 positively correlate with *PTTG3P*, *PTTG1* or *PTTG2*. The aforementioned genes interact with mutated and non-mutated p53. NF-Y influences the cell cycle progression, Pin1 promotes mutant p53 gain-of-function, and PML enhances transcriptional activity of mutant p53. Only p73 interacts with specific mutated variants of p53 and is responsible for regulation of the cell cycle and induction of apoptosis [23]. The group of negatively correlated genes comprises NF-YA (a member of NF-Y), Sp1, Ets-1, VDR, SREBP-2 and p63, which influence the cell cycle, drug resistance, promotion of cell survival and interaction with other genes from p53 pathway [23]. It should be noted that expression of p63 is reduced in advanced HNSCC, which results in metastasis promotion by activation of MAPK and STAT3 signaling pathways [35].

To further investigate the connection between the PTTG gene family and p53, we analyzed genes transcriptionally activated by mutant p53. The most positively correlated genes were found to be associated with increased proliferation (CCNB2, CDK1, CCNB1, STMN1, CDC25C), inhibition of apoptosis (NFKB2, TIMM50, DUT) and with centromere (CENPA, MIS18A). Whereas, the most negatively correlated are gene groups involved in the processes connected with increased proliferation (MYC, IGF1R, EGFR), metabolism (DHCR24, HMGCS1, SQLE, DHCR7, CYP51A1), cell–cell/cell–ECM signaling (ITGA6, MMP3, MMP13) and inhibition of apoptosis (IGF2). However, it should be noted that in the case of all three genes, *PTTG3P*, *PTTG1* and *PTTG2*, positive correlations were stronger than the negative ones. These findings indicate that higher expression of *PTTG3P, PTTG1* and *PTTG2* is connected with activation of prosurvival and protumorigenic downstream genes by mutant p53 protein. Indeed, our observations are supported by the results obtained by Read et al.; however, their analysis focused solely on *PTTG1* [34]. In our work, we separately examined the roles of *PTTG3P*, *PTTG1* and *PTTG2.* Similarly to Zhang et al., we observed a statistically significant positive correlation between *PTTG3P* and *PTTG1* as well as *PTTG2* in esophageal squamous cell carcinoma. The coexisting expression of *PTTG1* and *PTTG2* genes with the *PTTG3P* pseudogene may suggest that *PTTG3P* regulates *PTTG1* and *PTTG2*. It is possible that *PTTG3P* positively promotes the regulation of parental genes *PTTG1* and *PTTG2*, as is the case in esophageal squamous cell carcinoma [16]. In vitro studies have confirmed that exogenously the overexpression of *PTTG3P* promotes the transcription of its parental genes *PTTG1* and *PTTG2* in esophageal squamous cell carcinoma cells. However, the mechanism behind this discovery remains unknown [16].

Read et al. indicated that *PTTG1* together with PTTG1IP play an important role in regulation of p53 protein stability [34]. We observed correlation between all *PTTG3P, PTTG1* and *PTTG2* in HNSCC patients. Therefore, it comes as no surprise that in the case of p53 signaling pathway, we observed similar, but not identical, changes in gene expression for all members of the PTTG family. High expression of *PTTG1* was found to be associated with advanced cancers and short disease-free survival. Moreover Read et al., postulated that PTTG1IP does not reduce the stability of mutated p53 as it is observed in the case of mutant p53 protein in colorectal tumors [34]. The transcripts of *PTTG3P*, *PTTG1* and *PTTG2* seem to complement each other in fulfilling their biological function and influence on cellular program. However, this hypothesis should be verified using in vitro models.

Analysis of the relationship between *PTTG3P*, *PTTG1* and *PTTG2* expressions and clinicopathological characteristics in patients with HNSCCs revealed that high expression levels of *PTTG3P* correlated closely with lower T-stage, a higher grade and with HPV p16 status. As mentioned above, a higher expression of *PTTG3P* was observed in pharyngeal tumors with HPV status. This is additional evidence that *PTTG3P* may be involved in virus-related carcinogenesis in HNSCC. Roychowdhury et al. indicated that *PTTG3P*, as well as other 7 pseudogenes, are overexpressed in patient samples and cell lines of the uterine cervix carcinoma, where HPV infection is indicated as a primary causal agent [36]. Moreover, *PTTG3P* was correlated with a higher stage of uterine cervix carcinoma [36]. However, there is a lack of association studies of HNSCC, viral infection and *PTTG3P.* We also observed high expressions of *PTTG1* in higher cancer grade and in patients’ samples with negative perineural invasion, negative lymph node neck dissection and positive HPV p16 status. In contrast, *PTTG2* expression levels correlated with the N-stage of cancer, cancer grade, lymph node neck dissection, angiolymphatic invasion and HPV p16 status. The *PTTG* expression levels were previously found to correlate with clinicopathological parameters in HNSCCs, which was also the case in our analysis. Solbach et al. analyzed *PTTG* mRNA levels using northern blotting in 89 primary tumor samples and demonstrated a positive association between *PTTG* expressions and T and N tumor stages [37]. These findings support the results obtained in our analysis—higher *PTTG2* levels correlated with cancer N stage, whereas *PTTG3P* levels with the T stage of cancer. However, it should be noted that Solbach et al., in contrast to our findings, did not differentiate *PTTG* into three known transcripts, *PTTG3P*, *PTTG1* and *PTTG2* [37]. Other studies have shown that in esophageal cancer *PTTG* overexpression correlates with advanced pathological stage, extensive lymph node metastases and reduced survival [38]. *PTTG* expression in oropharyngeal tumors was significantly higher than at other subsites. A higher *PTTG1* expression significantly correlated with pathological stage and lymph node metastases [34,38]. Guo et al. indicated higher levels of *PTTG1* and *PTTG3P* in cervical cancer tissues compared to the paired adjacent healthy ones. Both were highly correlated and influenced by the EMT process and metastases [17]. Indeed, Zheng et al. revealed that FoxM1 transactivated *PTTG1* and promoted colorectal cancer cell migration and invasion by way of the regulation of the WNT pathway [39].

Our findings also support the observed association between *PTTG3P*, *PTTG1* and *PTTG2* with other genes. We indicated that many genes associated with important cellular processes are positively correlated with *PTTG3P*, *PTTG1* and *PTTG2*. It should be noted that among these genes are those connected with viral infections as well as tumor progression. It proves that *PTTG3P*, *PTTG1* and *PTTG2* may function as oncogenes in HNSCC, similarly as was indicated in other cancers [17,30,31,34,36,37,38,39,40].

This statement also confirms our observation that HNSCC patients with lower *PTTG3P* and *PTTG2* gene expressions exhibit longer DFS than patients in high expressing groups. Weng et al. demonstrated similar results in gastric cancer—high expressions of *PTTG3P* were correlated with significantly shorter DFS [40]. Even though our analysis did not provide evidence of *PTTG* genes’ expression associated with OS in HNSCC, there have been reports of such correlation in other cancer types [41]. Patients with non-small cell lung cancer with higher expression levels of the *PTTG1* gene exhibited shorter OS times [11]. However, Read et al. observed significant differences in the length of OS between *PTTG1* low and high expressing groups of HNSCC patients [34]. The reports mentioned above suggest that *PTTG* genes bear potential to become prognostic markers.

GSEA analysis indicated that patients with low expressions of pseudogene *PTTG3P*, as well as *PTTG1* and *PTTG2* genes have significantly negatively enriched genes involved in some pathways, such as DNA repair, E2F targets, Myc targets and peroxisome. These pathways are associated with carcinogenesis and cancer progression, which may explain that patients with low expressions of members of the *PTTG* family display better survival. However, in this group, the negative enrichment of genes connected with oxidative phosphorylation and G2M checkpoints was also observed.

DNA repair enzymes are responsible for the constant monitoring of chromosomes to correct damaged nucleotide residues induced in cells through external environmental factors. The result of DNA repair defects may be the accumulation of mutations that contribute to genomic instability and then to carcinogenesis [42]. The positive correlation of *PTTG* with E2F may be due to the fact that the cell cycle regulatory Rb-E2F pathway is a major target for HPV, and as we mentioned above, *PTTG* expression positively correlated with HPV. E2F family members are responsible for regulating the cell cycle during G1/S transition [42,43].

Moreover, we observed negative association of patients with low expression of *PTTG3P*, *PTTG1* as well as *PTTG2* genes and oxidative phosphorylation (OXPHOS). OXPHOS is a metabolic pathway that leads to the conversion of ADP to ATP. OXPHOS levels increase in cancer cells and form the major metabolic program in cancer initiating cells (cancer stem cells) [44,45]. Only for patients with low expression of *PTTG3P* was a negative association with peroxisome proliferator-activated receptor-γ (PPAR-γ) indicated in our study. The genes from this group participate in various aspects of cancer biology, including differentiation, proliferation, invasion and angiogenesis [46].

The next important pathways indicated by GSEA analysis are c-Myc targets, which are enriched in the group of patients with a higher level of *PTTG1*. In normal cells, the level of c-Myc protein is low. The level of c-Myc protein increases in cancer cells, especially in the final stages of cancer, because its expression is usually induced via upstream oncogenic pathways. In human cancer, an overexpression of c-Myc protein stimulates genes involved in protein biosynthesis, cancer metabolism, transcription factors and cell cycle genes [47]. This could potentially explain the observed worse patients’ disease-free survival and more advanced cancers (higher grade) in the group of patients with high levels in comparison to patients with low levels of *PTTG1.* However, negative enrichment of genes connected with the G2M checkpoint was observed by us in the case of patients with low expression levels of *PTTG2*. The G2M checkpoint is a particularly important cell cycle checkpoint because it ensures that cells do not initiate mitosis until the damaged or incompletely replicated DNA is fully repaired. G2M checkpoint abnormalities can lead to the proliferation of cells with damaged DNA [48]. It is difficult to explain why patients with a low expression of *PTTG2,* which is connected with better survival, have not got enriched genes connected with G2M checkpoint. However, it should be noted that in our analysis, we compared only cancer patients, so it is not surprising that some oncogenic pathways are up-regulated just as some pathways connected with tumor suppressors are down-regulated in the group of patients with better survival. We need to remember that patients with low expressions of *PTTG3P*, *PTTG1* and *PTTG2* have cancer. The comparison of data from healthy subjects with HNSCC patients divided into groups depending on expression level *PTTG3P*, *PTTG1* and *PTTG2* could give more sophisticated data about the role of these transcripts. A lack of full access to TCGA data makes our study limited to the presented results and conclusions. However, we are the first to not only thoroughly analyze the role of the pituitary tumor processing gene (*PTTG*) family, *PTTG3P*, *PTTG1* and *PTTG2*, in HNSCC, but we are also the first to indicate their important function as well as potential utility as biomarkers.

## 5. Conclusions

The major findings of the study are:

1. The expression of *PTTG3P*, *PTTG1* and *PTTG2* was upregulated in cancer tissue compared to normal in most cancer types including HNSCC and depends on TP53 status.
2. Statistical significance was obtained between tumors from the oral cavity and pharynx and between tumors from the pharynx and larynx in all three genes.
3. *PTTG3P* positively correlated with *PTTG1* and *PTTG2* in HNSCC tissue.
4. Patients with low expressions of *PTTG3P* and *PTTG2* displayed significantly longer disease-free survival.
5. Analysis of gene correlations and GSEA indicated that patients with high expressions of *PTTG3P*, *PTTG1* and *PTTG2* have different expression patterns of genes associated with carcinogenesis pathways in comparison to patients with low expressions of these three transcripts.

## Figures and Tables

**Table 1:** The expression levels of *PTTG3P*, *PTTG1* and *PTTG2* are associated with clinicopathological parameters in all localizations of HNSCC. *t*-test or Mann–Whitney U test; *p* < 0.05 considered as significant.

		*PTTG3P*			*PTTG1*			*PTTG2*		
Parameter	Group	Mean ± SEM	*p*-Value	*n*	Mean ± SEM	*p*-Value	*n*	Mean ± SEM	*p*-Value	*n*
**Grade**	G1 + G2	55.57 ± 2.016	<0.0001	368	1284 ± 58.95	<0.0001	368	11.17 ± 0.4642	0.005	368
	G3 + G4	73.03 ± 4.117		132	1623 ± 101.1		132	14.09 ± 0.9859		132
**HPV p16 status**	Negative	46.65 ± 3.023	<0.0001	80	958.6 ± 62.19	<0.0001	73	8.528 ± 0.6258	<0.0001	73
	Positive	102.6 ± 10.77		42	2601 ± 314.9		39	18.47 ± 1.983		39
**Lymph Node Neck Dissection**	Positive	59.05 ± 2.062	0.800	422	1382 ± 58.68	0.033	422	11.42 ± 0.4430	0.003	422
	Negative	68.07 ± 3.750		97	1420 ± 80.86		97	14.59 ± 1.133		97
**Angiolymphatic Invasion**	Positive	66.06 ± 4.830	0.154	125	1575 ± 132.8	0.098	125	13.56 ±1.061	0.034	125
	Negative	55.19 ± 2.428		225	1273 ± 72.55		225	10.61 ± 0.5364		225
**Perineural Invasion**	Positive	53.03 ± 2.570	0.557	169	1230 ± 75.92	0.032	167	10.97 ± 0.6415	0.210	169
	Negative	64.66 ± 3.580		195	1536 ± 102.5		193	12.42 ± 0.7516		195
**Age**	<60	62.39 ± 2.842	0.863	258	1454 ± 80.86	0.808	258	12.26 ± 0.6242	0.918	258
	>60	58.88 ± 2.276		263	1316 ± 58.67		263	11.82 ± 0.5682		263
**Gender**	Female	56.10 ± 3.600	0.077	137	1251 ± 94.08	0.055	137	10.98 ± 0.7738	0.070	137
	Male	62.25 ± 2.095		385	1434 ± 58.47		385	12.41 ± 0.4985		385
**Alcohol**	Positive	60.26 ± 2.235	0.494	348	1363 ± 58.02	0.606	348	11.59 ± 0.5001	0.151	348
	Negative	62.48 ± 3.258		163	1454 ± 99.58		163	13.06 ± 0.8033		163
**Smoking**	No/Ex	58.45 ± 2.628	0.911	191	1349 ± 60.24	0.711	294	11.74 ± 0.5441	0.708	294
	Yes	61.6 ± 2.487		319	1406 ± 82.29		216	12.07 ± 0.6676		216
**Cancer Stage**	I + II	55.40 ± 2.872	0.577	101	1154 ± 61.18	0.686	101	11.12 ± 0.8306	0.823	101
	III + IV	59.57 ± 2.371		349	1393 ± 66.51		349	11.93 ± 0.5197		349
**T Stage**	T1 + T2	63.94 ± 3.409	0.037	185	1434 ± 88.89	0.106	185	12.3 ± 0.7339	0.354	185
	T3 + T4	55.54 ± 2.266		274	1284 ± 65.23		274	11.48 ± 0.5600		274
**N Stage**	N0 + N1	56.15 ± 2.360	0.365	243	1266 ± 63.75	0.196	243	10.65 ± 0.5611	0.014	243
	N2 + N3	62.03 ± 3.618		179	1474 ± 100.9		179	12.99 ± 0.7680		179

## Supplementary Materials

The following are available online at https://www.mdpi.com/2075-4418/10/8/606/s1, Table S1: Correlation between the mutant p53-interacting partners and *PTTG3P*, *PTTG1* and *PTTG2*, Table S2: Correlation between transcriptionally activated genes by mutant p53 protein and *PTTG3P*, *PTTG1* and *PTTG2*, Table S3: HNSCC patients’ survival depending on the low and high expression of mutant p53-interacting partners, Table S4: HNSCC patients’ survival depending on the low and high expression of transcriptionally activated genes by mutant p53 protein, Table S5: List of positively and negatively correlated genes with *PTTG3P*, *PTTG1* or *PTTG2* and involved in important cellular processes, Table S6: List of protein coding genes in the pathways which were the most significantly enriched in a group of patients with high expression of *PTTG3P*, *PTTG1* or *PTTG2*.

## Abbreviations

BLCA	Bladder urothelial carcinoma
BRCA	Breast invasive carcinoma
CESC	Cervical squamous cell carcinoma
CHOL	Cholangiocarcinoma
COAD	Colon adenocarcinoma
CT	Chemotherapy
DFS	Disease-free survival
ESCA	Esophageal carcinoma
GBM	Glioblastoma multiforme
GSEA	Gene Set Enrichment Analysis
HNSCC	Head and Neck squamous cell carcinoma
HPV	Human papillomavirus
KICH	Kidney Chromophobe
KIRC	Kidney renal clear cell carcinoma
KIRP	Kidney renal papillary cell carcinoma
LIHC	Liver hepatocellular carcinoma
LUAD	Lung adenocarcinoma
LUSC	Lung squamous cell carcinoma
OS	Overall survival
OXPHOS	Oxidative phosphorylation
PAAD	Pancreatic adenocarcinoma
PRAD	Prostate adenocarcinoma
RT	Radiotherapy
SARC	Sarcoma
SKCM	Skin Cutaneous Melanoma
TCGA	The Cancer Genome Atlas
THCA	Thyroid carcinoma
THYM	Thymoma
STAD	Stomach adenocarcinoma
UCEC	Uterine Corpus Endometrial Carcinoma
