# Supplementary material for: The Oncogenic Roles of PTTG1 and PTTG2 Genes and Pseudogene PTTG3P in Head and Neck Squamous Cell Carcinomas
Source: Diagnostics (Basel). 2020 Aug 18;10(8):606. doi: 10.3390/diagnostics10080606 (PMC7459614; doi:10.3390/diagnostics10080606)
Supplement: Supplementary file 1 [file diagnostics-10-00606-s001.pdf]

Supplementary Table S1. Correlation between the mutant p53-interacting partners and *PTTG3P, PTTG1 and PTTG2*, based on data from StarBase v3.0 database.

Gene	ID	*PTTG3P*		*PTTG1*		*PTTG2*	
		**Coefficient-R**	***p*-value**	**Coefficient-R**	***p*-value**	**Coefficient-R**	***p*-value**
NF-YA	ENSG00000001167	−0.077	8.59e-2	−0.210	2.09e-6	−0.122	6.23e-3
NF-YB	ENSG00000120837	0.176	7.12e-5	0.227	2.82e-7	0.094	3.59e-2
NF-YC	ENSG00000066136	0.124	5.45e-3	0.124	5.40e-3	0.051	2.51e-1
Sp1	ENSG00000185591	−0.014	7.50e-1	−0.201	5.82e-6	−0.072	1.07e-1
Ets-1	ENSG00000134954	−0.096	3.14e-2	−0.257	4.83e-9	0.034	4.46e-1
VDR	ENSG00000111424	−0.091	4.10e-2	−0.216	1.03e-6	0.014	7.48e-1
SREBP-2	ENSG00000198911	−0.064	1.53e-1	−0.147	9.27e-4	−0.073	1.01e-1
TopBP1	ENSG00000163781	0.067	1.36e-1	0.051	2.57e-1	−0.020	6.57e-1
Pin1	ENSG00000127445	0.250	1.40e-8	0.571	9.56e-45	0.187	2.52e-5
MRE11	ENSG00000020922	0.063	1.56e-1	−0.007	8.81e-1	−0.024	5.93e-1
PML	ENSG00000140464	0.072	1.05e-1	0.217	9.36e-7	0.166	1.85e-4
p63	ENSG00000073282	−0.120	7.04e-3	−0.283	1.08e-10	−0.198	7.71e-6
p73	ENSG00000078900	0.104	2.03e-2	0.258	4.67e-9	0.097	3.02e-2

Supplementary Table S2. Correlation between transcriptionally activated genes by mutant p53 protein and *PTTG3P, PTTG1* and *PTTG2*, based on data from StarBase v3.0 database.

Gene	ID	*PTTG3P*		*PTTG1*		*PTTG2*	
		**Coefficient-R**	***p*-value**	**Coefficient-R**	***p*-value**	**Coefficient-R**	***p*-value**
MYC	ENSG00000136997	−0.046	3.06e-1	−0.178	6.13e-5	−0.196	1.01e-5
CXCL1	ENSG00000163739	−0.087	5.26e-2	0.048	2.79e-1	0.007	8.84e-1
MAP2K3	ENSG00000034152	−0.047	2.88e-1	−0.058	1.92e-1	0.069	1.20e-1
FOS	ENSG00000170345	−0.100	2.56e-2	−0.154	5.16e-4	−0.120	6.94e-3
PCNA	ENSG00000132646	0.038	3.99e-1	0.548	1.04e-40	0.183	3.71e-5
MAD1L1	ENSG00000002822	0.154	5.55e-4	0.275	3.78e-10	0.071	1.10e-1
CCNE2	ENSG00000175305	0.066	1.41e-1	0.013	7.77e-1	−0.081	6.98e-2
CCNA2	ENSG00000145386	0.206	3.24e-6	0.370	9.85e-18	0.039	3.87e-1
CCNB1	ENSG00000134057	0.319	2.33e-13	0.619	2.09e-54	0.132	3.14e-3
CCNB2	ENSG00000157456	0.296	1.31e-11	0.579	3.36e-46	0.085	5.74e-2
CDK1	ENSG00000170312	0.299	7.36e-12	0.562	3.65e-43	0.157	4.02e-4
CDC25C	ENSG00000158402	0.375	3.34e-18	0.722	4.65e-82	0.241	4.32e-8
E2F5	ENSG00000133740	0.089	4.74e-2	0.142	1.40e-3	0.118	8.07e-3
ASNS	ENSG00000070669	0.097	2.92e-2	0.161	2.82e-4	0.024	5.94e-1
IGF1R	ENSG00000140443	−0.145	1.14e-3	−0.323	1.14e-13	−0.132	2.99e-3
IGFBP3	ENSG00000146674	−0.059	1.86e-1	−0.085	5.59e-2	−0.012	7.87e-1
EGFR	ENSG00000146648	−0.145	1.10e-3	−0.335	1.37e-14	−0.213	1.47e-6
MCM6	ENSG00000076003	0.241	4.43e-8	0.351	4.83e-16	0.095	3.31e-2
STMN1	ENSG00000117632	0.351	5.86e-16	0.577	7.77e-46	0.216	1.09e-6
EGR1	ENSG00000120738	−0.071	1.12e-1	−0.136	2.28e-3	−0.096	3.10e-2
NFKB2	ENSG00000077150	0.181	4.32e-5	0.374	4.59e-18	0.323	1.25e-13
ABCB1	ENSG00000085563	−0.084	6.07e-2	−0.180	5.12e-5	0.070	1.17e-1
TIMM50	ENSG00000105197	0.244	2.90e-8	0.484	7.49e-31	0.136	2.21e-3
LGALS3	ENSG00000131981	0.059	1.84e-1	0.133	2.80e-3	0.026	5.67e-1
BCL2L1	ENSG00000171552	0.051	2.54e-1	0.110	1.35e-2	0.062	1.67e-1
IGF2	ENSG00000167244	−0.170	1.30e-4	−0.424	2.73e-23	−0.157	4.28e-4
DUT	ENSG00000128951	0.324	1.05e-13	0.641	1.60e-59	0.232	1.50e-7
ACAT2	ENSG00000120437	0.084	6.14e-2	0.165	2.05e-4	0.029	5.10e-1
HMGCS1	ENSG00000112972	−0.097	2.96e-2	−0.293	2.01e-11	−0.143	1.29e-3
HMGCR	ENSG00000113161	0.082	6.64e-2	−0.182	4.09e-5	−0.101	2.40e-2
MVK	ENSG00000110921	0.097	3.04e-2	0.264	1.88e-9	0.022	6.21e-1
PMVK	ENSG00000163344	0.080	7.28e-2	0.327	5.98e-14	0.085	5.76e-2

MVD	ENSG00000167508	0.007	8.81e-1	0.144	1.17e-3	0.005	9.03e-1
IDI1	ENSG00000067064	0.030	5.04e-1	0.078	8.08e-2	−0.049	2.78e-1
FDPS	ENSG00000160752	0.099	2.64e-2	0.192	1.44e-5	−0.010	8.30e-1
FDFT1	ENSG00000079459	0.089	4.62e-2	0.116	9.19e-3	−0.039	3.78e-1
SQLE	ENSG00000104549	−0.098	2.78e-2	-0.249	1.64e-8	−0.135	2.35e-3
LSS	ENSG00000160285	0.000	9.98e-1	-0.029	5.12e-1	−0.044	3.20e-1
CYP51A1	ENSG00000001630	−0.188	2.24e-5	−0.441	2.63e-25	−0.239	5.86e-8
TM7SF2	ENSG00000149809	−0.079	7.70e-2	−0.094	3.59e-2	−0.040	3.69e-1
SC4MOL	ENSG00000224339	0.039	3.80e-1	0.034	4.44e-1	0.092	3.97e-2
NSDHL	ENSG00000147383	0.076	8.99e-2	0.242	3.96e-8	−0.019	6.66e-1
DHCR7	ENSG00000172893	−0.101	2.37e-2	−0.118	8.29e-3	−0.135	2.39e-3
DHCR24	ENSG00000116133	−0.082	6.76e-2	−0.149	8.10e-4	−0.084	6.05e-2
CYP24A1	ENSG00000019186	0.015	7.38e-1	0.015	7.33e-1	0.088	4.82e-2
MMP3	ENSG00000149968	−0.183	3.57e-5	−0.291	3.19e-11	−0.077	8.66e-2
MMP13	ENSG00000137745	−0.193	1.29e-5	−0.272	5.45e-10	−0.067	1.34e-1
ITGA6	ENSG00000091409	−0.136	2.23e-3	−0.285	8.20e-11	−0.191	1.69e-5
PXN	ENSG00000089159	−0.025	5.79e-1	−0.135	2.48e-3	−0.059	1.84e-1
ARHGDIA	ENSG00000141522	−0.026	5.62e-1	−0.043	3.33e-1	−0.092	3.97e-2
RANGAP1	ENSG00000100401	0.110	1.34e-2	0.377	2.14e-18	0.166	1.94e-4
DEPDC1	ENSG00000024526	0.164	2.33e-4	0.262	2.39e-9	−0.008	8.51e-1
WDR67	ENSG00000253410	−0.106	1.78e-2	−0.136	2.28e-3	−0.049	2.76e-1
TERT	ENSG00000164362	0.251	1.20e-8	0.294	1.68e-11	0.199	6.84e-6
KIF20A	ENSG00000112984	0.237	7.55e-8	0.332	2.19e-14	0.103	2.06e-2
EPB41L4B	ENSG00000095203	−0.041	3.60e-1	−0.153	5.58e-4	−0.142	1.46e-3
BUB1	ENSG00000169679	0.162	2.68e-4	0.263	2.29e-9	0.002	9.56e-1
NCAPH	ENSG00000121152	0.149	8.03e-4	0.241	4.63e-8	0.037	4.02e-1
MIS18A	ENSG00000159055	0.335	1.22e-14	0.547	1.65e-40	0.163	2.56e-4
CENPA	ENSG00000115163	0.256	6.04e-9	0.474	1.59e-29	0.083	6.26e-2
CPSF6	ENSG00000111605	0.161	3.03e-4	0.134	2.70e-3	0.021	6.39e-1
FAM64A	ENSG00000129195	0.185	3.15e-5	0.381	8.77e-19	0.091	4.13e-2
ID4	ENSG00000172201	−0.088	4.84e-2	−0.088	4.79e-2	−0.012	7.85e-1

Supplementary Table S3. HNSCC patients' survival depending on the low and high expression of mutant p53-interacting partners, based on data from StarBase v3.0 database.

**Gene**	**ID**	**Cases**	**Median**	**Coef**	**HR**	***p*-value**

NF-YA	ENSG00000001167	495	7.88	−0.12	0.89	0.38
NF-YB	ENSG00000120837	495	4.31	−0.22	0.81	0.12
NF-YC	ENSG00000066136	495	7.69	−0.05	0.95	0.72
Sp1	ENSG00000185591	495	13.64	−0.03	0.97	0.85
Ets-1	ENSG00000134954	495	11.74	−0.17	0.84	0.21
VDR	ENSG00000111424	495	10.75	0.15	1.16	0.28
SREBP-2	ENSG00000198911	495	30.79	−0.04	0.96	0.76
TopBP1	ENSG00000163781	495	8.75	−0.05	0.95	0.69
Pin1	ENSG00000127445	495	7.96	−0.31	0.73	0.023
MRE11	ENSG00000020922	495	2.66	−0.16	0.85	0.24
PML	ENSG00000140464	495	13.29	0.09	1.10	0.49
p63	ENSG00000073282	495	64.81	−0.00	1.00	1
p73	ENSG00000078900	495	3.42	−0.28	0.76	0.042

Supplementary Table S4. HNSCC patients' survival depending on the low and high expression of transcriptionally activated genes by mutant p53 protein, based on data from StarBase v3.0 database.

Gene	ID	Cases	Median	coef	HR	*p*-value
MYC	ENSG00000136997	495	64.63	0.25	1.29	0.065
CXCL1	ENSG00000163739	495	27.06	0.07	01.08	0.59
MAP2K3	ENSG00000034152	495	19.80	0.08	01.08	0.57
FOS	ENSG00000170345	495	86.40	−0.18	0.84	0.19
PCNA	ENSG00000132646	495	86.65	−0.05	0.95	0.7
MAD1L1	ENSG00000002822	495	5.47	−0.02	0.98	0.87
CCNE2	ENSG00000175305	495	01.06	−0.17	0.84	0.2
CCNA2	ENSG00000145386	495	14.31	0.10	1.10	0.48
CCNB1	ENSG00000134057	495	30.00	−0.04	0.96	0.76
CCNB2	ENSG00000157456	495	13.69	−0.03	0.97	0.82
CDK1	ENSG00000170312	495	12.38	−0.02	0.98	0.91
CDC25C	ENSG00000158402	495	1.68	0.15	1.16	0.27
E2F5	ENSG00000133740	495	0.62	−0.02	0.98	0.87
ASNS	ENSG00000070669	495	5.94	0.23	1.26	0.097
IGF1R	ENSG00000140443	495	09.09	0.31	1.36	0.026
IGFBP3	ENSG00000146674	495	41.52	0.02	01.02	0.9
EGFR	ENSG00000146648	495	23.91	0.20	1.22	0.15
MCM6	ENSG00000076003	495	15.63	−0.08	0.92	0.56
STMN1	ENSG00000117632	495	26.73	0.07	01.08	0.59
EGR1	ENSG00000120738	495	63.90	−0.05	0.95	0.72
NFKB2	ENSG00000077150	495	15.37	0.05	01.05	0.74
ABCB1	ENSG00000085563	495	0.22	−0.48	0.62	0.0004
TIMM50	ENSG00000105197	495	08.04	0.04	01.04	0.76
LGALS3	ENSG00000131981	495	78.20	−0.30	0.74	0.031

BCL2L1	ENSG00000171552	495	27.99	0.05	01.05	0.74
IGF2	ENSG00000167244	495	6.40	0.09	1.10	0.5
DUT	ENSG00000128951	495	13.08	0.03	01.03	0.82
ACAT2	ENSG00000120437	495	08.07	0.01	01.01	0.94
HMGCS1	ENSG00000112972	495	19.75	0.23	1.26	0.097
HMGCR	ENSG00000113161	495	12.57	0.04	01.04	0.79
MVK	ENSG00000110921	495	4.32	−0.23	0.79	0.09
PMVK	ENSG00000163344	495	37.97	−0.12	0.89	0.4
MVD	ENSG00000167508	495	10.24	0.17	1.19	0.21
IDI1	ENSG00000067064	495	14.36	0.16	1.18	0.24
FDPS	ENSG00000160752	495	25.84	0.06	01.06	0.66
FDFT1	ENSG00000079459	495	20.94	−0.11	0.90	0.43
SQLE	ENSG00000104549	495	29.15	0.44	1.55	0.0015
LSS	ENSG00000160285	495	9.62	−0.13	0.88	0.35
CYP51A1	ENSG00000001630	495	1.37	0.11	1.11	0.44
TM7SF2	ENSG00000149809	495	4.15	−0.18	0.84	0.19
SC4MOL	ENSG00000224339	495	0.00	0.00	0.00	1
NSDHL	ENSG00000147383	495	21.29	0.14	1.15	0.31
DHCR7	ENSG00000172893	495	20.40	0.28	1.33	0.042
DHCR24	ENSG00000116133	495	117.19	0.01	01.01	0.94
CYP24A1	ENSG00000019186	495	3.20	−0.19	0.83	0.16
MMP3	ENSG00000149968	495	22.32	0.12	1.13	0.36
MMP13	ENSG00000137745	495	09.04	0.12	1.12	0.39
ITGA6	ENSG00000091409	495	117.73	0.17	1.19	0.2
PXN	ENSG00000089159	495	21.09	0.28	1.33	0.039
ARHGDIA	ENSG00000141522	495	97.30	0.06	01.07	0.65
RANGAP1	ENSG00000100401	495	47.15	0.11	1.11	0.44
DEPDC1	ENSG00000024526	495	2.87	0.16	1.17	0.24
WDR67	ENSG00000253410	495	0.00	0.00	0.00	1
TERT	ENSG00000164362	495	0.10	−0.19	0.83	0.16
KIF20A	ENSG00000112984	495	9.53	0.10	1.11	0.45
EPB41L4B	ENSG00000095203	495	2.46	−0.10	0.90	0.45
BUB1	ENSG00000169679	495	5.36	0.15	1.16	0.29
NCAPH	ENSG00000121152	495	7.34	0.14	1.15	0.29
MIS18A	ENSG00000159055	495	7.60	0.10	1.10	0.47
CENPA	ENSG00000115163	495	5.74	−0.01	0.99	0.93
CPSF6	ENSG00000111605	495	9.88	−0.02	0.98	0.89
FAM64A	ENSG00000129195	495	3.29	0.10	1.11	0.45
ID4	ENSG00000172201	495	2.82	−0.02	0.98	0.86

Supplementary Table S5. List of positively and negatively correlated genes with *PTTG3P, PTTG1 or PTTG2* involved in important cellular processes.

Analyzed Gene	Type of Correlation	Process	Genes
PTTG3P	Positive	Cell cycle	SMC3, SMC4, SMC2, CDC20, PSMD9, CDC23, RCC1, CCDC99, FBXO5, GTSE1, TMPO, SKP1, ESCO2, C16orf75, PSME1, PSME2, KIF20A, DNA2, TP53, PIF1, RTEL1, SET, ANAPC16, CDCA5, NCAPG, CDCA8, AAAS, SKA1, NCAPH, SKA2, DSN1, BRIP1, NUP85, C14orf93, ATRIP, CEP78, PLK4, CDT1, NCAPH2, PLK1, CDC7, CDC6, ZWINT, DHFR, POLA1, TPX2, POLA2, RAD17, TOP2A, LIN54, FEN1, ORC1L, MLF1IP, NCAPG2, BRCA1, BRCA2, TUBA1B, XPO1, NUF2, UIMC1, TK1, NINL, NUDC, RMI1, KIF23, TUBG1, PSMA5, RAD51C, PSMA4, KIF2A, PSMA2, DMC1, KIF2C, SFI1, PRIM2, PCNA, PRIM1, SSNA1, TYMS, CENPA, PSMC3IP, PPP2CA, PSMB7, PSMB4, RAD54L, CDKN2D, GINS1, MPP2, GINS2, NPM1, CDKN2C, CENPW, CDKN2A, GINS3, GINS4, RPA2, MLH1, PSMB9, NEDD1, CENPE, PSMC5, CENPF, APITD1, PSMC3, CENPH, CENPI, RPA3, CENPJ, CENPK, C21orf45, CENPL, CENPM, CENPN, B9D2, NCAPD3, CENPO, CENPQ, C12orf32, SPC24, MAD2L1, SPC25, ERCC6L, ZWILCH, DSCC1, GMNN, HJURP, BUB1B, PTTG1, PTTG2, RUVBL2, KNTC1, OIP5, NEK2, AZI1, CEP135, LIG1, VRK1, VRK2, CDC25C, CDC25A, CDC25B, CCNE2, NHP2, BLM, ORC3L, HMMR, PKMYT1, PMF1, CCNB2, CCNB1, CLSPN, CEP110, UBE2C, NDC80, KIF18A, UBE2S, CDK4, CDK2, CDK1, FGFR1OP, RAD9B, WRAP53, C19orf62, MCM7, MCM8, MCM10, FOXM1, CHTF18, CKS1B, LMNB1, SGOL2, SGOL1, EXO1, NUP62, MYBL2, WHSC1, POLE, RFC5, RFC3, RFC4, RFC1, H2AFZ, RFC2, H2AFX, H2AFV, HAUS5, MASTL, HAUS1, CCNA2, DBF4, ESPL1, MCM3, BIRC5, MCM4, MCM5, MCM6, ITGB3BP, MCM2, LIN9, AURKB, NSL1, PSMB10, AURKA, POLD3, CDC45, RBBP4, HAUS8, HAUS7, POLD1, E2F1, E2F2, NUP43, RBBP7, BUB3, POLR2I, BUB1, POLR2K, RRM2, CEP152, ORC6L, MND1, RAD51, POLE2
		Gene expression	CCNH, TACO1, UBE2D2, C1orf59, EHMT1, TTF1, COX6A1, UXT, PSMD9, LSM11, CDC23, TRIM28, RBMX2, MAGOH, C12orf62, SKP1, ZNF684, LIFR, THOC3, CDC25C, KCTD6, THOC7, C16orf75, SAP30, MED7, MSH2, CCNE2, FANCD2, SARNP, PSME1, PSME2, SNRPF, DNA2, TP53, SNRPB, SLBP, DNMT1, BLM, ANAPC16, C1orf77, CSTF3, SUPT4H1, AAAS, OSGEP, SFRS7, CCNB1, BRIP1, NUP85, PMAIP1, C14orf93, ATRIP, NR2C2AP, FANCI, PRELID1, SURF1, CBX5, UBE2C, CPSF3, UPF3B, FANCC, CNOT10, CDC7, SFRS2, SMARCA2, ESR1, SFRS3, C5orf35, TPX2, MOV10, CNOT6, UBE2S, CDK4, CDK2, CDK1, RAD17, PIN1, CALM3, CNOT8, RAD9B, ZNF530, TP73, EZH2, PHF19, COX7B, PHF1, SUV39H1, DPY30, BRCA1, COX7C, CASP6, EXO1, NUP62, CASP2, MYBL2, MYBL1, COX8A, RFC5, RFC3, RFC4, LRDD, RMI1, H2AFZ, PRMT1, RFC2, H2AFX, H2AFV, MED27, DDB2, CCNA2, PSMA5, OBFC2B, PSMA4, ZNF519, PSMA2, ZNF879, MTF2, BIRC5, BDP1, SLU7, ZNF473, PCNA, HDAC3, GTF3C5, GLS2, UHRF1, SRRT, TAF9, U2AF1, COX5A, AURKB, PSMB10, HDAC6, AURKA, PPP2CA, PSMB7, PSMB4, RBBP4, PABPN1, SAP30L, TIFA, E2F1, SAP18, E2F2, NUP43, RBBP7, POLR2I, E2F7, POLR2K, E2F8, MBD3, DDX39, NPM1, PCGF6, RRM2,

		CDKN2A, ATAD2, RPA2, DEK, MLH1, TARBP2, PSMB9, PSMC5, RAD51, PSMC3, RPA3, CENPJ, CCNG1, FAS, TAF7, BAX, TAF5, RHEBL1, C12orf32
	Metabolism of RNA	RPP30, CWC27, THG1L, GLE1, RPS14, PSMD9, FBL, LSM11, SNRPD2, SNRPD1, MAGOH, TXNL4A, CDKAL1, THOC3, ZCRB1, THOC7, SARNP, NHP2, PSME1, PPIE, PSME2, PPIH, SNRPF, SNRNP25, SNRPC, SNRPA, SNRPB, SF4, SLBP, SET, C1orf77, CSTF3, AAAS, RNPC3, OSGEP, SFRS7, NUP85, ERI1, SMN2, C14orf93, RBM10, ZRSR2, PRPF38A, APOBEC3H, CPSF3, UPF3B, CNOT10, SFRS2, SFRS3, LSM3, LSM2, HNRNPL, HNRNPM, CNOT6, LSM6, LAS1L, HNRNPD, HNRNPC, CNOT8, APOBEC3B, RPS23, NXT1, C19orf61, PPWD1, PQBP1, XPO1, NUP62, BCAS2, UTP15, PSMA5, QTRT1, PSMA4, SYF2, HNRNPH1, PSMA2, DNAJC8, SLU7, SKIV2L2, DCP2, RBM22, TSEN34, ZNF473, URM1, SRRT, DDX20, RPS27L, SFRS13A, NOC4L, U2AF1, HSD17B10, PSMB10, PPP2CA, PSMB7, EXOSC5, PSMB4, EXOSC10, PABPN1, EXOSC9, EXOSC8, TIFA, NUP43, POLR2I, HNRNPA1, EXOSC2, RPL17, EXOSC1, POLR2K, HNRNPA0, SF3A3, DDX39, SF3A2, GPKOW, TPRKB, PSMB9, SNRNP40, PSMC5, PSMC3, HNRNPA2B1, PRPF31, B9D2, PPIL3, RBMX
	DNA repair	C19orf62, FEN1, DCLRE1C, WDR48, CCNH, OGG1, BRCA1, BRCA2, ALKBH2, EME1, EXO1, UIMC1, SUMO2, POLL, WHSC1, KPNA2, POLE, POLH, HMGN1, GEN1, RFC5, TIPIN, RFC3, RFC4, RFC1, LIG1, H2AFZ, RMI1, RFC2, H2AFX, H2AFV, C16orf75, DDB2, MSH6, CCNA2, NEIL3, RAD51C, MSH2, MSH3, FANCD2, TIMELESS, PPIE, DNA2, TP53, DTL, C19orf40, RTEL1, INO80E, BLM, PCNA, UBA7, PNKP, UNG, RAD51AP1, POLD3, BRIP1, POLD1, USP1, CLSPN, POLR2I, ATRIP, POLR2K, MUTYH, FANCI, POLQ, XRCC4, EYA2, FANCL, MCRS1, FANCA, XRCC1, RPA2, FANCC, FANCB, MLH1, FANCG, MAD2L2, COPS3, PPP5C, APITD1, RAD51, KIAA0101, RPA3, POLE2, UBE2T, CDK2, POLE3, CDK1, ERCC8, RAD17, RAD9B, C12orf32
	DNA replication	ORC1L, MCM7, MCM8, GMNN, MCM10, PSMD9, CDC23, POLE, SKP1, RFC5, RFC3, RFC4, RFC1, LIG1, RFC2, PSMA5, CCNA2, PSMA4, DBF4, CCNE2, PSMA2, MCM3, PSME1, MCM4, PSME2, MCM5, DNA2, MCM6, MCM2, PRIM2, PCNA, ANAPC16, ORC3L, PRIM1, PSMB10, POLD3, PSMB7, PSMB4, CDC45, POLD1, E2F1, E2F2, C14orf93, GINS1, CDT1, GINS2, UBE2C, GINS3, GINS4, ORC6L, RPA2, CDC7, CDC6, PSMB9, POLA1, PSMC5, POLA2, PSMC3, UBE2S, RPA3, POLE2, CDK2, POLE3, CDK1
	Organelle biogenesis and maintenance	ATP5G2, ATP5G1, HSPB11, TUBA1B, CHCHD6, NEK2, NINL, AZI1, CEP135, KIF24, HAUS5, TUBG1, ATP5F1, WDR34, HAUS1, SFI1, HDAC3, ARL3, SSNA1, C1orf151, ATP5J, ATP5H, ATP5O, HDAC6, APOO, HAUS8, HAUS7, PDE6D, CCT8, CYC1, CEP78, PLK4, HSPA9, CEP110, PHF10, CEP152, PLK1, WDR19, SCLT1, IMMT, IFT122, NEDD1, CENPJ, CDK1, FGFR1OP, CALM3, DYNLRB1
	Chromatin organization	SUV420H2, SUV39H2, HDAC3, KDM3B, SUV39H1, TAF9, EHMT1, DPY30, NSL1, ING4, RBBP4, RUVBL2, SAP30L,

			SAP18, RBBP7, DMAP1, BRD8, WHSC1, C12orf41, MBD3, H2AFZ, PRMT1, MCRS1, H2AFX, H2AFV, C3orf75, ELP4, SMARCA2, NFKB2, SAP30, CDK4, PHF17
		Programmed cell death	HMGB2, HMGB1, PSMB10, LMNB1, PSMD9, PSMB7, PSMB4, CASP6, E2F1, E2F2, PMAIP1, C14orf93, CLSPN, DFFB, TICAM2, CDKN2A, GZMB, TRAF2, GZMH, PSMB9, PSMA5, PSMC5, PSMA4, PSMC3, PSMA2, BCL2, PSME1, BAX, FAS, PSME2, TP53, DCP2, TP73
		HIV viral infection	CCNH, TAF9, SUPT4H1, PSIP1, AAAS, PSMB10, PSMD9, PSMB7, PSMB4, XPO1, NUP85, NUP62, RCC1, C14orf93, NUP43, POLR2I, POLR2K, SKP1, NPM1, XRCC4, LIG1, APOBEC3G, PSMB9, PSMA5, PSMC5, PSMA4, CD8B, PSMC3, PSMA2, PSME1, TAF7, PSME2, TAF5, SLC25A5
	Negative	Developmental biology processes (keratinization)	PERP, PKP1, SPRR1A, SPRR1B, IVL, KRT6A, SPRR2D
		Signal transduction	SMOX, USP2, PKP1, PTK6, SDR9C7, NDEL1
PTTG1	Positive	Cell cycle	CCNH, ZWILCH, DSCC1, GMNN, HJURP, BUB1B, SMC4, SMC2, CDC20, PSMD9, CDC23, PTTG2, RUVBL2, CHEK2, STRA13, KNTC1, OIP5, NEK2, CCDC99, FBXO5, GTSE1, AZI1, TMPO, SKP1, LIG1, VRK1, ESCO2, CDC25C, CDC25A, C16orf75, RBX1, CDC25B, NHP2, PSME1, PSME2, KIF20A, PIF1, BLM, RTEL1, ANAPC16, PSMD13, CDCA5, ORC3L, NCAPG, CDCA8, HMMR, PKMYT1, PMF1, SKA1, SKA2, ANAPC11, CCNB2, CCNB1, DSN1, NUP85, GAR1, ATRIP, CEP78, PLK4, CDT1, CEP110, UBE2C, NCAPH2, H3F3A, CDC7, NDC80, ZWINT, DHFR, POLA1, TPX2, POLA2, KIF18A, UBE2S, CDK4, CDK2, CHMP2A, CDK1, RAD17, RAD9B, RAD9A, WRAP53, C19orf62, FEN1, ORC1L, MCM7, MLF1IP, NCAPG2, BRCA1, CHTF18, HIST2H2AC, CKS1B, LMNB1, TUBA1B, SGOL1, EXO1, NUF2, MYBL2, TK1, WHSC1, NINL, EMD, POLE, RFC5, NUDC, RFC3, RFC4, RMI1, H2AFZ, RFC2, H2AFX, H2AFV, KIF23, HAUS5, RANGAP1, HAUS1, CCNA2, PSMA5, RAD51C, PSMA4, PSMA2, MCM3, BIRC5, KIF2C, MCM5, MCM6, SFI1, ITGB3BP, MCM2, PCNA, PRIM1, SSNA1, TYMS, HDAC8, CENPA, AURKB, NSL1, PSMB10, AURKA, PSMC3IP, PSMB6, PSMB7, PSMB4, CDC45, HAUS8, PSMB3, HAUS7, POLD1, E2F1, RAD54L, RBBP7, BUB3, POLR2H, POLR2I, POLR2K, CDKN2D, GINS1, MPP2, GINS2, NPM1, CDKN2C, CENPW, RRM2, CDKN2A, CEP152, GINS4, RPA2, ORC6L, MND1, PPP2R3B, MLH1, FAM128B, FAM128A, PSMB9, POLE4, PSMC5, RAD51, APITD1, PSMC3, CENPH, RPA3, CENPJ, POLE2, CENPK, C21orf45, POLE3, CENPM, CENPN, B9D2, SPC24, MAD2L1, SPC25
		Metabolism of RNA	RPL32, CCNH, RPL34, RPP30, RRP1, CWC27, THG1L, RPS15, RPS14, PSMD9, FBL, RPS19, SNRPD2, SNRPD1, MAGOH, RPL35, SNRPD3, SNRNP35, RPS11, TXNL4A, RPL39, RPS9, RPS8, RPL22, RPSA, THOC3, THOC4, THOC7, SF3B14, SARNP, RPP21, NHP2, PSME1, RPL24, PPIE, PSME2, SNRPE, PPIH, SNRPF, GEMIN7, SNRNP25, SNRPC, SNRPA, SNRPB, SF4, SLBP, C1orf77, PSMD13, CSTF3, OSGEP, SFRS7, NUP85, GAR1, RBM10, ZRSR2, RPL41, APOBEC3H, CPSF3, UPF3B, CNOT10, MAGOHB, LSM5, LSM4, SFRS3, LSM3, LSM2, ISG20,

		HNRNPL, HNRNPM, LSM7, LSM6, QARS, LAS1L, RPS20, CNOT8, RPS24, APOBEC3B, RPS23, DDX49, NXT1, C19orf61, PPWD1, PQBP1, SNRNP70, PSMA5, QTRT1, PSMA4, SYF2, HNRNPH1, PSMA2, DNAJC8, SLU7, DCP2, RBM22, TSEN34, ZNF473, BAT1, URM1, SRRT, RPL11, RPS27L, NOC4L, U2AF1, HSD17B10, LAGE3, PSMB10, PSMB6, REM1, PSMB7, EXOSC5, PSMB4, PABPN1, PSMB3, EXOSC9, EXOSC8, RPL15, POLR2H, RPL18, HNRNPA1, POLR2I, EXOSC2, RPL17, EXOSC1, POLR2K, HNRNPA0, SF3A3, DDX39, GPKOW, TPRKB, PSMB9, PSMC5, PSMC3, PRPF31, B9D2, RBMX, RPL26L1
	Cellular responses to external stimuli	UBE2D2, RPL34, RRP1, NUDT2, TCIRG1, HIST2H2AC, LMNB1, RPS15, RPS14, PSMD9, TUBA1B, ROBLD3, CDC23, RPS19, GPBP1, RPL35, RPS11, RPL39, RPS9, GPX1, H2AFZ, RPS8, H2AFX, RPL22, H2AFV, COMMD3, RPSA, ETV2, RBX1, EEF1A1, PSMA5, CCNA2, PSMA4, DNAJC7, PSMA2, PSME1, RPL24, PSME2, ANAPC16, RPS19BP1, PSMD13, NPRL2, RPL11, RPS27L, PSMB10, ANAPC11, PSMB6, HBXIP, PSMB7, PRDX2, PSMB4, NUP85, PSMB3, E2F1, TCEB2, RPL15, RBBP7, RPL18, RPL17, CDKN2D, HSPA9, RPL41, CDKN2C, CDKN2A, UBE2C, H3F3A, RPA2, TBCB, EIF2S2, ATOX1, SOD1, PSMB9, PSMC5, PSMC3, CDK4, UBE2S, RPA3, CDK2, CDK1, RPS20, RHEBL1, RPS24, RPL26L1, EZH2, RPS23
	DNA repair	FEN1, DCLRE1C, CCNH, OGG1, RRP1, BRCA1, HIST2H2AC, ALKBH2, EME1, EXO1, CHEK2, SUMO2, POLL, STRA13, WHSC1, POLE, HMGN1, RFC5, TIPIN, RFC3, RFC4, LIG1, H2AFZ, RMI1, RFC2, H2AFX, H2AFV, SIRT6, C16orf75, RBX1, DDB2, CCNA2, NEIL3, RAD51C, FANCD2, TIMELESS, PPIE, DTL, RTEL1, INO80E, BLM, PCNA, PNKP, RAD51AP1, REM1, POLD1, USP1, POLR2H, POLR2I, ATRIP, POLR2K, MUTYH, FANCI, POLQ, XRCC4, MGMT, EYA2, FANCL, MCRS1, FANCA, XRCC1, RPA2, FANCC, FANCB, MLH1, FANCG, GIYD2, POLE4, MAD2L2, PPP5C, APITD1, RAD51, KIAA0101, RPA3, POLE2, UBE2T, CDK2, POLE3, CDK1, RAD17, RAD9B, RAD18, RAD9A
	DNA replication	ORC1L, MCM7, GMNN, PSMD9, CDC23, POLE, SKP1, RFC5, RFC3, RFC4, LIG1, RFC2, RBX1, PSMA5, CCNA2, PSMA4, PSMA2, MCM3, PSME1, PSME2, MCM5, MCM6, MCM2, PCNA, ANAPC16, PSMD13, ORC3L, PRIM1, PSMB10, ANAPC11, PSMB6, PSMB7, PSMB4, CDC45, PSMB3, POLD1, E2F1, GINS1, CDT1, GINS2, UBE2C, GINS4, ORC6L, RPA2, CDC7, PSMB9, POLE4, POLA1, PSMC5, POLA2, PSMC3, UBE2S, RPA3, POLE2, CDK2, POLE3, CDK1
	Programmed cell death	DIABLO, HMGB2, HMGB1, PSMB10, LMNB1, PSMB6, PSMD9, PSMB7, PSMB4, CASP6, PSMB3, E2F1, PMAIP1, DFFB, CDKN2A, BAD, GZMB, TRAF2, GZMH, PSMB9, PSMA5, PSMC5, PSMA4, PSMC3, PSMA2, PSME1, BAX, FAS, PSME2, DCP2, TP73, BIRC3
	Protein localization	FH, NDUFB8, TIMM16, UBE2D2, COX17, TIMM10, HSCB, TAZ, ATP5G1, CHCHD4, HMGCL, TIMM17B, HACL1, SEC61B, CYC1, EMD, HSPA9, ACOT8, CAMLG, IDH3G, CHCHD10, GFER, COQ2, EIF3K, PXMP2, C18orf55, SLC25A5, MTCP1NB, ACOT4

		Viral infection (HIV and influenza)	PSMD13, CCNH, TAF9, PSIP1, PSMB10, PSMB6, REM1, PSMD9, PSMB7, PSMB4, PSMB3, NUP85, TCEB2, POLR2H, POLR2I, POLR2K, SKP1, NPM1, XRCC4, LIG1, APOBEC3G, RDBP, RANGAP1, RBX1, PSMB9, PSMA5, PSMC5, PSMA4, CD8B, PSMC3, PSMA2, PSME1, CHMP2A, PSME2, SLC25A5
	Negative	Immune system	PGLYRP3, IRS1, RAB3D, CLTC, WASL, NPEPPS, PPP2R1B, CDH1, AP1G1, KIF5B, TOM1, QSOX1, LRRFIP1, KPNA3, IL6R, DSP, ACTR3, MAP2K4, ACTR2, TRIM62, GAB1, UBE4A, LRRC41, ENAH, ACLY, STIM1, IL1F5, TMBIM1, MYH9, DSG1, PKP1, PAFAH1B2, IL28RA, C16orf62, DYNC1I2, SEC23A, CAMK2D, NLRX1, CUL5, RASAL2, NOD2, PSEN1, HIF1A, PPL, PLD2, RNF217, MAPK3, VAT1, DYNC1H1, UBE2H, JUP, MYO5A, IGF2R, RAB10, SDC1, TRIP12, CALM1, EIF4G3
		Developmental biology	FURIN, WASL, PSEN1, EVPL, PPL, ABLIM1, SPTLC3, PERP, IL6R, KRT6C, MYH10, KRT6B, KRT6A, MAPK3, SPRR2D, ACTR3, DSP, SPAG9, ACTR2, ARHGEF12, JUP, GAB1, ENAH, EFNA3, MAFB, MYH9, DSG1, PKP1, DSG3, SPRR1A, SPRR1B, DSC2, IVL, CDK5R1
		Vesicle-mediated transport	DYNC1I2, SEC23A, CLTC, WASL, CYTH3, RABGEF1, GJA1, AP1G1, KIF5B, KIF1C, KIF1B, TBC1D16, SH3D19, ACTR3, ACTR2, DYNC1H1, DENND2C, MYO5A, IGF2R, RAB10, GJB2, KIF26B, MYH9, TRIP11, CALM1, PAFAH1B2
		Programmed cell death	CDH1, DSG1, PKP1, DSG3, MAPK3
		Cell–cell communication	JUP, CDH1, FBLIM1, WASL, PVRL1
PTTG2	Positive	Cell cycle	ERCC6L, ZWILCH, GMNN, HJURP, BUB1B, SMC4, SMC2, CDC20, CDC23, PTTG1, RUVBL2, KNTC1, OIP5, NEK2, FBXO5, CCDC99, GTSE1, TMPO, NUP214, LIG1, CDC25C, CDC25A, C16orf75, NHP2, KIF20A, DNA2, BLM, ANAPC16, CDCA5, NCAPG, CDCA8, HMMR, PKMYT1, AAAS, PMF1, SKA1, SKA2, CCNB2, CCNB1, BRIP1, CLSPN, CEP78, PLK4, CDT1, CEP110, UBE2C, RCC2, PLK1, CDC7, ZWINT, DHFR, POLA1, TPX2, POLA2, KIF18A, CDK2, CDK1, RAD17, RAD9B, WRAP53, TOP2A, FEN1, ORC1L, MCM7, MLF1IP, NCAPG2, BRCA1, FOXM1, CKS1B, LMNB1, TUBA1B, SGOL2, SGOL1, EXO1, UIMC1, NUP62, MYBL2, TK1, WHSC1, POLE, RFC5, RFC3, RFC4, RFC1, H2AFZ, RFC2, RMI1, H2AFV, KIF23, HAUS5, HAUS1, CCNA2, RAD51C, ESPL1, MCM3, KIF2C, MCM5, MCM6, ITGB3BP, MCM2, PRIM2, PCNA, PRIM1, LIN9, TYMS, CENPA, NSL1, AURKB, AURKA, PSMC3IP, HAUS8, POLD1, E2F2, RAD54L, BUB3, RBBP7, BUB1, GINS1, GINS2, NPM1, RRM2, CDKN2C, CDKN2A, CEP152, GINS3, RPA2, MND1, CENPF, RAD51, APITD1, CENPH, CENPI, RPA3, POLE2, CENPJ, POLE3, CENPK, C21orf45, CENPL, CENPM, B9D2, CENPO, C12orf32, SPC24, SPC25, MAD2L1
		Gene expression (transcription)	SUV39H1, UBE2D2, BRCA1, COX7C, LSM11, CDC23, TRIM28, EXO1, NUP62, CASP2, MYBL2, MYBL1, NUP214, RFC5, RFC3, RFC4, H2AFZ, LRDD, RFC2, RMI1, H2AFV, THOC3, CDC25C, C16orf75, MED7, DDB2, SAP30, CCNA2, OBFC2B, FANCD2, ZNF879, DNA2, SLU7, ZNF473, SLBP, DNMT1, BLM, PCNA,

			HDAC3, ANAPC16, GLS2, UHRF1, TAF9, U2AF1, AAAS, AURKB, OSGEP, AURKA, SFRS7, BRIP1, CCNB1, SAP30L, E2F2, RBBP7, E2F7, E2F8, FANCI, WWOX, NPM1, PCGF6, RRM2, CBX5, CDKN2A, UBE2C, ATAD2, RPA2, FANCC, CDC7, DEK, MOV10, TPX2, CNOT6, RAD51, RPA3, CENPJ, CCNG1, CDK2, CDK1, FAS, RAD17, CALM3, CNOT8, RAD9B, RHEBL1, C12orf32, PHF19, TP73, EZH2
		DNA, repair	MDC1, FEN1, DCLRE1C, BRCA1, EME1, EXO1, UIMC1, WHSC1, POLE, POLH, HMGN1, GEN1, RFC5, RFC3, RFC4, RFC1, LIG1, H2AFZ, RFC2, RMI1, H2AFV, C16orf75, DDB2, MSH6, CCNA2, NEIL3, RAD51C, MSH3, FANCD2, TIMELESS, DNA2, DTL, BLM, PCNA, PNKP, UNG, BRIP1, POLD1, USP1, CLSPN, FANCI, POLQ, FANCL, FANCA, XRCC1, RPA2, FANCC, FANCB, FANCG, PPP5C, APITD1, RAD51, KIAA0101, RPA3, POLE2, UBE2T, CDK2, POLE3, CDK1, RAD17, RAD9B, C12orf32, RAD18
		Metabolism of RNA	ZNF473, SLBP, URM1, RPP30, PPWD1, U2AF1, AAAS, CWC27, OSGEP, THG1L, GLE1, SFRS7, EXOSC5, LSM11, EXOSC9, NUP62, HNRNPA1, RBM10, EXOSC1, HNRNPA0, APOBEC3C, SF3A3, NUP214, SF3A2, GPKOW, FUS, APOBEC3H, THOC3, HNRNPL, HNRNPM, CNOT6, HNRNPUL1, NHP2, PPIH, PRPF31, B9D2, SNRPC, CNOT8, SLU7, RBMX, SNRPA, DCP2, RPL26L1, APOBEC3B, RBM22
		DNA replication	FEN1, PCNA, ORC1L, MCM7, ANAPC16, PRIM1, GMNN, CDC23, POLD1, E2F2, POLE, GINS1, RFC5, CDT1, GINS2, RFC3, RFC4, RFC1, LIG1, UBE2C, RFC2, GINS3, RPA2, CDC7, CCNA2, POLA1, POLA2, RPA3, POLE2, CDK2, POLE3, MCM3, CDK1, MCM5, DNA2, MCM6, MCM2
		Organelle biogenesis and maintenance	PLK4, HDAC3, CEP110, CEP152, ARL3, PLK1, SCLT1, IMMT, KIF24, HAUS5, WDR34, HSPB11, HAUS1, APOO, TUBA1B, HAUS8, CHCHD6, CENPJ, CDK1, NEK2, CALM3, B9D2, CEP78
	Negative	Developmental biology processes (keratinization)	SPRR1B, IVL

Supplementary Table S6. List of protein coding genes in the pathways which were the most significantly enriched in a group of patients with high expression of *PTTG3P, PTTG1* or *PTTG2*. Only results set with $p \leq 0.05$ and FDR ≤ 0.25 were listed.

Analyzed Gene	Process	Enriched Genes in a Group of Patients with High Expression of Analyzed Gene
PTTG3P	Peroxisome	ABCC5, YWHAH, DHCR24, CTBP1, ALB, MSH2, PRDX1, ERCC3, ESR2, ERCC1, TSPO, SLC25A17, SLC25A19, PRDX5, CDK7, HRAS, EPHX2, SOD1
	DNA repair	SMAD5, ERCC2, ERCC5, GTF2F1, POLB, MPG, ERCC3, TSG101, TP53, ERCC1, GPX4, CCNO, DDB2, RAD51, POLL, FEN1, LIG1, GTF2B, PCNA, NME1, RPA3, ADA, ERCC8
	Oxidative phosphorylation	CASP7, MTX2, ETFA, AIFM1, SLC25A5, RHOT2, GRPEL1, TIMM17A, HSPA9, TIMM8B, GPX4, SLC25A20, PRDX3, TIMM50, TIMM9, VDAC1, BAX, TIMM10,

		TOMM22, SLC25A3, CYCS
PTTG1	DNA repair	ADA, POLD3, ERCC1, ERCC8, GTF2F1, GPX4, MPG, CCNO, GTF2B, NME1, POLL, DDB2, LIG1, PCNA, RAD51, FEN1, RPA3
	MYC targets	HSPD1, COPS5, CDK2, CCT3, PRDX4, MCM2, CCT4, SET, KPNA2, HNRNPA2B1, RPS6, MCM5, CCNA2, CCT7, MAD2L1, SLC25A3, CDK4, PTGES3, HSPE1, PPIA, HNRNPD, NME1, VDAC1, PCNA, RUVBL2, NHP2, CDC20
	Oxidative phosphorylation	LDHA, ETFA, GPI, TIMM8B, MGST3, TOMM22, CYCS, SLC25A5, GPX4, TIMM9, AIFM1, HSPA9, TIMM17A, TIMM10, SLC25A3, TIMM50, RHOT2, VDAC1, BAX
	E2F targets	MLH1, TOP2A, MKI67, DCLRE1B, BRCA1, XRCC6, UNG, CDKN2A, PRDX4, MCM2, CHEK2, KPNA2, PSIP1, MCM5, PLK1, MAD2L1, PLK4, H2AFX, CDK4, CDC25A, CDKN2C, AURKA, MCM3, HNRNPD, NME1, LMNB1, BIRC5, LIG1, PCNA, CDKN3, STMN1, RPA3, RAD51C, CKS2, CDK1, CDC20, CCNB2, AURKB, KIF2C, PTTG1
PTTG2	Oxidative phosphorylation	TIMM17A, CASP7, RHOT2, GRPEL1, COX10, LDHA, TIMM8B, MGST3, TOMM22, GPX4, CYCS, SLC25A3, GPI, SLC25A5, AIFM1, TIMM9, TIMM10, TIMM50, HSPA9, VDAC1, BAX
	E2F targets	XRCC6, TP53, MLH1, POLD3, CHEK2, TOP2A, CBX5, UNG, PRDX4, RFC1, DCLRE1B, MAD2L1, CDK4, CDKN2A, BRCA1, MCM2, PLK1, H2AFX, PSIP1, KPNA2, PLK4, AURKA, MKI67, NME1, CDC25A, MCM5, CKS2, RAD51C, PCNA, STMN1, BIRC5, MCM3, CDKN2C, LMNB1, CDK1, LIG1, CDC20, KIF2C, CDKN3, RPA3, CCNB2, AURKB, PTTG1
	G2M checkpoint	CDK4, MCM2, E2F1, PLK1, EWSR1, H2AFX, KPNA2, EXO1, PLK4, PRC1, CCNA2, AURKA, MKI67, CDC25A, MCM5, CKS2, STMN1, RAD54L, BIRC5, CCNF, MCM3, CDKN2C, LMNB1, CDK1, CDC20, KIF2C, CDKN3, CCNB2, AURKB, PTTG1
